# Two new species of *Dugesia* (Platyhelminthes, Tricladida, Dugesiidae) from the subtropical monsoon region in Southern China, with a discussion on reproductive modalities

**DOI:** 10.1186/s40850-022-00127-8

**Published:** 2022-05-23

**Authors:** Guang-wen Chen, Lei Wang, Fan Wu, Xiao-juan Sun, Zi-mei Dong, Ronald Sluys, Fei Yu, Yan-qing Yu-wen, De-zeng Liu

**Affiliations:** 1 College of Life Science, Henan Normal University, Xinxiang, 453007 Henan China; 2 Medical College, Xinxiang University, Xinxiang, 453003 China; 3 Naturalis Biodiversity Center, Leiden, The Netherlands

**Keywords:** Genetic distance, Karyology, Molecular phylogeny, New species, Reproduction, Taxonomy

## Abstract

**Background:**

Freshwater planarians of the genus *Dugesia* (Platyhelminthes, Tricladida, Dugesiidae) are distributed in a major part of the Old World and Australia, although until recently only very few species were known from China.

**Results:**

Two new species of *Dugesia* from Southern China are described on the basis of an integrative taxonomic approach. BI and ML phylogenetic trees based on the independent genes and on the concatenated dataset had similar topologies, only differing in some nodes that were weakly supported. Phylogenetic trees based on the concatenated dataset revealed that *D. adunca* Chen & Sluys, sp. nov. and *D. tumida* Chen & Sluys, sp. nov. are not closely related and belong to different clades. The two new species occupy separate long branches with high support values and, thus, are well-differentiated from their congeners. Separate species status of *D. adunca* and *D. tumida* is supported also by the genetic distances between the species included in our analysis, albeit that COI distances varied greatly among species. *Dugesia adunca* from Guangxi Province is characterized by the following features: living mature animals rather small; asymmetrical openings of the oviducts into the bursal canal; penis papilla with shape of an aquiline bill, albeit with a blunt tip; asymmetrical penis papilla, with a large antero-dorsal lip and a much smaller ventro-posterior lip; very large seminal vesicle, provided with trabeculae; small diaphragm; mixoploid karyotype with diploid complements of 2n = 2x = 16 and triploid complements of 2n = 3x = 24, with all chromosomes being metacentric. *Dugesia tumida* from Guangdong Province is characterized by a penis papilla provided with a large, symmetrical penial valve from the middle of which arises the small, distal section of the papilla; a duct intercalated between the seminal vesicle and the small diaphragm; ventrally displaced ejaculatory duct curving upwards before opening to the exterior; penis papilla highly asymmetrical, having a slim and long ventral portion and a short and stubby dorsal part; vasa deferentia separately opening into antero-dorsal portion of seminal vesicle; oviducts openings symmetrically into ventral portion of the bursal canal, near its opening into the atrium; mixoploid karyotype, with diploid chromosome portraits of 2n = 2x = 16, and triploid complements of 2n = 3x = 24, with all chromosomes being metacentric. In the context of the various kinds of mixoploidy and the sexualization of specimens, reproductive modalities within the genus *Dugesia* are shortly discussed.

**Conclusion:**

Molecular, morphological, and karyological markers show that the two populations examined represent members of the genus *Dugesia* and constitute two new, distinct species.

**Supplementary Information:**

The online version contains supplementary material available at 10.1186/s40850-022-00127-8.

## Background

Freshwater planarians of the genus *Dugesia* Girard, 1850 are among the best known triclad flatworms because of their characteristic triangular head with prominent auricles, carrying two eyes that are set in pigment-free spots, and notably for their great capacity for regeneration. Although this regeneration capacity is undiminished in species of *Dugesia*, it turned out that the few model species that are generally used in laboratories are not members of this genus but do actually belong to several different genera, as is the case with the species *Schmidtea mediterranea* (Benazzi et al., 1975), *S. lugubris* (Schmidt, 1861), and *Girardia tigrina* (Girard, 1850), all of which were formerly assigned to *Dugesia*.

About 100 species of *Dugesia* have been reported from the major portion of the Old World and Australia, but it was only recently that taxonomic studies started to discover the rich biodiversity of this genus in China. In particular, recent taxonomic studies revealed that Southern China harbours several, previously unknown species of *Dugesia* Girard, 1850 (e.g. [1–4]). This may be attributed to the tropical monsoon climate of Southern China, which contributes to the rich biodiversity of this climatic region of the world [5–7]. In this paper, we describe two new species of *Dugesia* from Southern China, in particular from Guangxi Province and Guangdong Province, based on an integrative taxonomic approach, including morphological, histological, karyological and molecular data. In the general discussion section we pay attention to the various kinds of mixoploidy and the sexualization of specimens that may occur in species of *Dugesia* and how that may affect reproductive modalities within the genus.

## Methods

### Specimen collection and culturing

Specimens were collected on the Shiwan Dashan Mountain, Guangxi Province and the Yunwu Mountain, Guangdong Province. Worms were taken, with the help of a paint brush, from the underside of stones in a stream or pond, and then transferred to plastic bottles filled with stream water that during transportation to the laboratory were placed in a cooler filled with an ice bag. The planarians were cultured under semi-dark conditions in glass beakers filled with autoclaved tap water at 20 °C, and were fed with fresh beef liver once per week. Worms that were used for karyotype examination, histological studies, and DNA extraction were starved for at least one week. Images of their external features were obtained by using a digital camera attached to a stereo dissecting microscope. Colour descriptions of the body of the worms follow online RAL palette colours (© RAL gemeinnützige GmbH, available at https://www.ral-farben.de/en/all-ral-colours).

### DNA extraction, amplification, sequencing, phylogenetic analysis, and genetic distances

Procedures for DNA extraction, amplification and sequencing followed those detailed in Wang et al. [3, 4]. Fragments of the Cytochrome c oxidase subunit I (COI) and internal transcribed spacer-1 (ITS-1) were amplified using the primer pairs BarS & COIR and 9F & ITSR, respectively (e.g. [8–11]). For each of the two new species, four specimens were used to extract DNA, from which COI and ITS-1 were amplified.

In total, 67 sequences of 36 species (two new *Dugesia* species, 31 congeners, and three outgroup species) were used to perform phylogenetic analyses and to calculate genetic distances, with *Schmidtea mediterranea*, *S. polychroa* (Schmidt 1861), and *Recurva postrema* Sluys & Solà, 2013 constituting the outgroup taxa (for GenBank accession numbers, see Table 1).

**Table 1:** GenBank accession numbers of COI and ITS-1 sequences used for molecular analyses

Species	GenBank	
	CO I	ITS-1
*D. adunca*	OL505739*	OL527659*
*D. aethiopica*	KY498845	KY498785
*D. afromontana*	KY498846	KY498786
*D. arcadia*	KC006971	KC007044
*D. ariadnae*	KC006972	KC007048
*D. batuensis*	KF907818	KF907815
*D. benazzii*	FJ646933^a^ + FJ646977^b^	FJ646890
*D. bengalensis*	-	FJ646897
*D. bifida*	KY498851	KY498791
*D. circumcisa*	MZ147041	MZ146782
*D. cretica*	KC006976	KC007050
*D. constrictiva*	MZ871766	MZ869023
*D. deharvengi*	KF907820	KF907817
*D. elegans*	KC006984	KC007063
*D. etrusca*	FJ646939^a^ + FJ646984^b^	FJ646898
*D. gibberosa*	KY498857	KY498803
*D. gonocephala*	FJ646941^a^ + FJ646986^b^	FJ646901
*D. hepta*	FJ646943^a^ + FJ646988^b^	FJ646902
*D. japonica*	FJ646990 AB618487	FJ646904
*D. liguriensis*	FJ646992	FJ646907
*D. majuscula*	MW533425	MW533591
*D. naiadis*	KF308756	-
*D. notogaea*	FJ646945^a^ + FJ646993^b^	FJ646908
*D. ryukyuensis*	AF178311 AB618488	FJ646910
*D. sagitta*	KC007006	KC007077
*D. semiglobosa*	MW525210	MW526992
*D. sicula*	FJ646947^a^ + FJ646994^b^	DSU84356
*D. sigmoides*	KY498849	KY498789
*D. sinensis*	KP401592	-
*D. subtentaculata*	FJ646949^a^ + FJ646995^b^	DSU84369
*D. tumida*	OL505740*	OL527709*
*D. umbonata*	MT176641	MT177211
*D. verrucula*	MZ147040	MZ146760
*R. postrema*	KF308763	-
*S. mediterranea*	JF837062	AF047854
*S. polychroa*	FJ646975^a^ + FJ647021^b^	-

In Lázaro et al. [8] study, the COI makers are amplified by two pairs primers (COIs and COIbc), thus a short fragment and a long fragment are obtained from one sample, respectively

^a^ sequences amplified by COIs

^b^ sequences amplified by COIbc

^*^ New sequences

The method of sequences preparation was described previously by Wang et al. [3, 4]. In brief, ITS-1 sequences were aligned online with MAFFT (Online Version 7.247) using the G-INS-i algorithm [12]. With respect to COI sequences, Translator X (http://translatorx.co.uk) was used under FFT-NS-2 method [13], and were checked by BioEdit 7.2.6.1 [14, 15]. The concatenated sequences for the phylogenetic analysis consisted of a total of 1385 base pairs (bp) with the order of ITS-1 + COI.

The best-fit partition schemes and models of the concatenated sequences were estimated by applying the Bayesian information criterion (BIC) implemented in PartitionFinder 2 [16, 17]. The selected models for each gene and codon position were GTR + G for ITS-1 and first codon position of COI, HKY + I + G for the second and third codon position of COI. Bayesian inference analysis (BI) was run with MrBayes v 3.2 [18] for each gene independently, as well as for the concatenated dataset. We ran the analyses using two replicate runs with four chains for 5 million generations and sampled trees every 1000 generations. The average standard deviation of split frequencies was lower than 0.01, indicating that the runs had reached stationarity. Following completion of each analysis, the first 25% of the generated trees was discarded as “burn-in”, while the remaining trees were used to generate a consensus phylogram and to calculate statistics for the taxon bipartitions, values for clade support (posterior probability) and branch lengths. Maximum-likelihood (ML) analysis with RaxML 8.2.10 [19] was used to perform 1,000 replicates under the GTRGAMMA model. BI and ML trees were visualized and edited using Figtree v1.4.3.

MEGA 6.06 [20] was used to calculate genetic distances of COI and ITS-1 under the Kimura 2-parameter substitution model [8, 21]. Since the research of Marques et al. [22] indicated that at least 600 bp of COI need to be used in the determination of interspecific divergences and for species delineation, at least in the land planarian genus *Obama* Carbayo et al., 2013, we removed COI sequences that had less than 600 bp, in order to obtain more accurate values for the genetic distances.

### Histology and karyology

Histological sections were prepared as described previously by Dong et al. [23]. In brief, worms were fixed in Bouin’s fluid for 24 h, and thereafter, rinsed and stored in 70% ethanol. For histological study, specimens were dehydrated in an ascending series of ethanol solutions, then cleared in clove oil and embedded in synthetic paraffin. Serial sections were made at intervals of 6 μm and were stained with hematoxylin–eosin, or in hematoxylin and Cason's Mallory-Heidenhain stain [24]. Photomicrographs were taken with a Leica digital camera attached to a compound microscope. Histological preparations of specimens have been deposited in the Zoological Museum of the College of Life Science of Henan Normal University (ZMHNU), Xinxiang, China, and Naturalis Biodiversity Center, Leiden, The Netherlands (RMNH).

The air-drying method was used to obtain karyological preparations, according to protocols detailed in Dong et al. [23] and Wang et al. [3, 4]. The mitotic metaphase chromosomes were photographed and examined under a compound microscope equipped with a digital camera. In order to determine ploidy level, centromeric indices, and relative lengths of the chromosomes, at least 10 well-spread sets of metaphase plates from five or six randomly selected individuals were used to analyse the karyotype, of which parameter measurements were determined as detailed previously by Chen et al. [25]. Chromosomal nomenclature follows Levan et al. [26].

## Results

### Molecular phylogeny and genetic distances

The concatenated sequences are composed of COI sequences (750 base pairs—bp) and ITS-1 sequences (635 bp). The four COI sequences amplified from four specimens of *Dugesia adunca* Chen & Sluys, sp. nov. were checked by BioEdit 7.2.6.1, and exhibited no variation. The same result was found for the four ITS-1 sequences of *D. adunca*, the four COI sequences of *D. tumida* Chen & Sluys, sp. nov., and the four ITS-1 sequences of *D. tumida*.

**Fig. 1:**
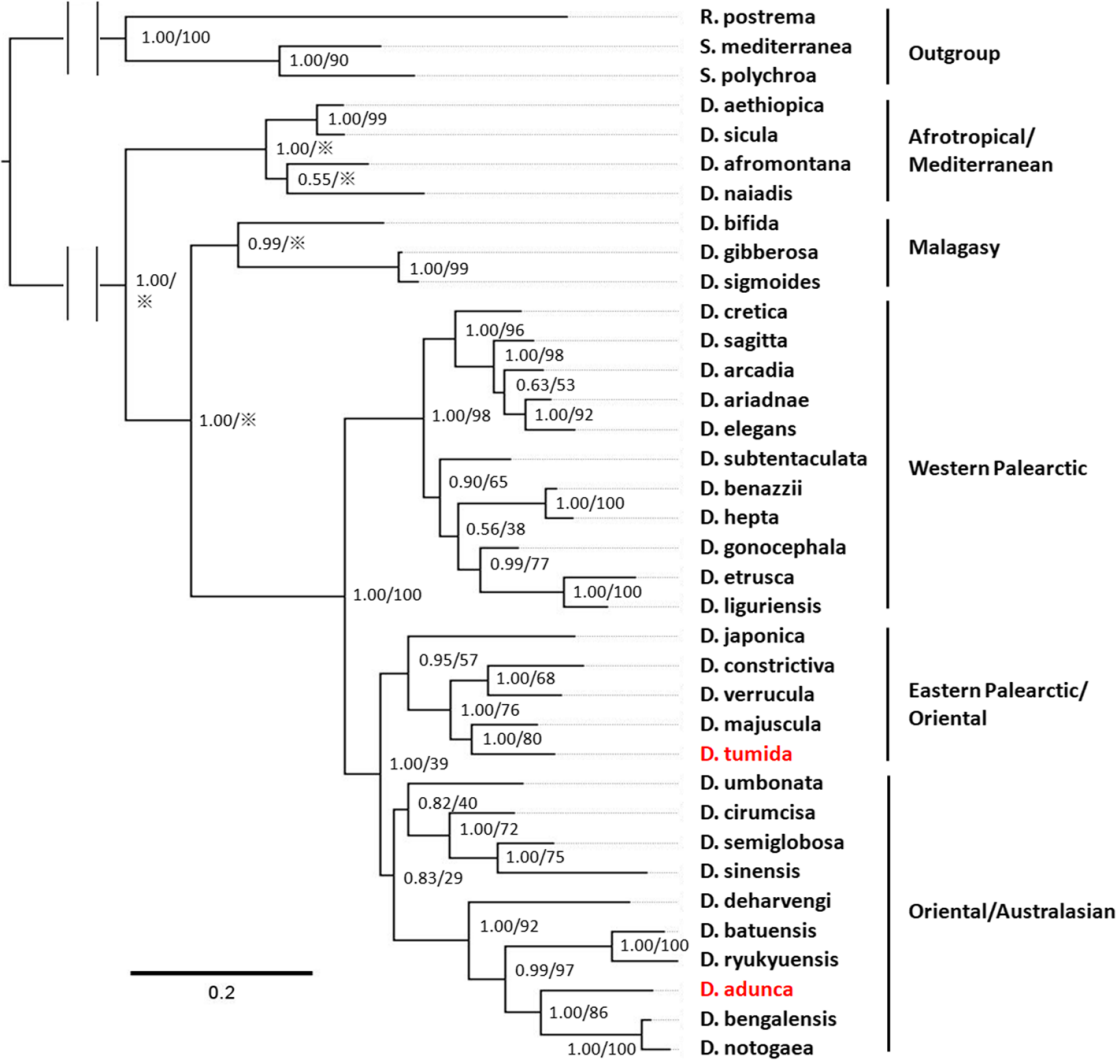
Molecular phylogenetic tree obtained from Bayesian analysis of the concatenated dataset. Numbers at nodes indicate support values (pp/bs).*: Bootstrap value not applicable to the node, because of different topologies of trees obtained by BI and ML methods. New species indicated in red. Scale bar: substitutions per site

The BI and ML phylogenetic trees based on the independent genes and concatenated dataset had similar topologies, only differing in some nodes that were weakly supported (Fig. 1; Supporting Information Fig. S1, S2 and S3). Numbers at nodes indicate support, posterior probability (pp) in BI trees, and bootstrap (bs) in ML tree.

With respect to the phylogenetic trees based on the concatenated dataset (Fig. 1 and Supporting Information Fig. S1), it is noteworthy that *D. adunca* and *D. tumida* are not closely related and belong to different clades. In the Eastern Palearctic/Oriental group, *D. tumida* shares a sister-group relationship with *D. majuscula* Chen & Dong, 2021 (with high support values: pp = 1.00, bs = 80), and together belong to a clade that includes *D. verrucula* Chen & Dong, 2021, *D. constrictiva* Chen & Dong, 2022 and *D. japonica* Ichikawa & Kawakatsu, 1964. *Dugesia adunca* is a part of one major clade (with high support values: pp = 1.00, bs = 86) within the Oriental/Austral-asian group that comprises also the species *D. deharvengi* Kawakatsu & Mitchell, 1989, *D. batuensis* Ball, 1970, D*. ryukuyensis* Kawakatsu, 1976, *D. bengalensis* Kawakatsu, 1983, and *D. notogaea* Sluys & Kawakatsu, 1998.

With respect to the trees generated from the independent genes (Supporting Information Fig. S2, S3), it should be noted that the COI sequence of *D. bengalensi*s Kawakatsu, 1983 and the ITS-1 sequence of *D. sinensis* Chen & Wang, 2015 are absent, which results in slightly different positions for the two new species, as compared with the tree based on the concatenated dataset. However, the BI tree of COI still shows a high support value for the sister-group relationship of *D. tumida* and *D. majuscula* (pp = 1.00). Concerning the BI tree of ITS-1, the *D. adunca* terminal belongs to the same monophyletic group as compared with tree based on the concatenated dataset (high support value, pp = 1.00).

In conclusion, all phylogenetic trees reveal that the two new species occupy separate long branches with high support values (Fig. 1; Supporting Information Fig. S1, S2 and S3) and, thus, that they are separate species that are well-differentiated from their congeners.

The separate species status of *D. adunca* and *D. tumida* is supported also by the genetic distances between the species included in our analysis, albeit that COI distances vary greatly among species (Supporting Information Table S1). The highest distance value between *D. adunca* and its congeners is 22.86% (with *D. sicula* Lepori, 1948), while the lowest distance value is 14.15% (with *D. notogaea* Sluys & Kawakatsu, 1998). With respect to *D. tumida*, the highest distance value between this species and its congeners is 24.76% (with *D. sicula*), while the lowest distance value is 14.71% (with *D. majuscula* and *D. verrucula*). Furthermore, there is a 20.80% difference between the two new species.

With respect to ITS-1, *D. adunca* and *D. tumida* show highest distance values with *D. sicula*, which are 20.69% and 16.12%, respectively. Furthermore, *D. adunca* shows the lowest distance value with *D. bengalensis*, which is 6.14%, while for *D. tumida* the lowest distance value is with *D. majuscula*, viz., 2.21%. For the ITS-1 marker, the molecular distance between the two new species is 9.77% (Supporting Information Table S2).

### Systematic Account

Order Tricladida Lang, 1884

Suborder Continenticola Carranza, Littlewood, Clough, Ruiz-Trillo, Baguñà & Riutort, 1998

Family Dugesiidae Ball, 1974

Genus *Dugesia* Girard, 1850

***Dugesia adunca*** **Chen & Sluys, sp. nov.**

Zoobank registration: LSID: urn:lsid:zoobank.org:act:11333F07-B91A-4610–9714-804FD083BF20.

### Collection site and habitat

**Fig. 2:**
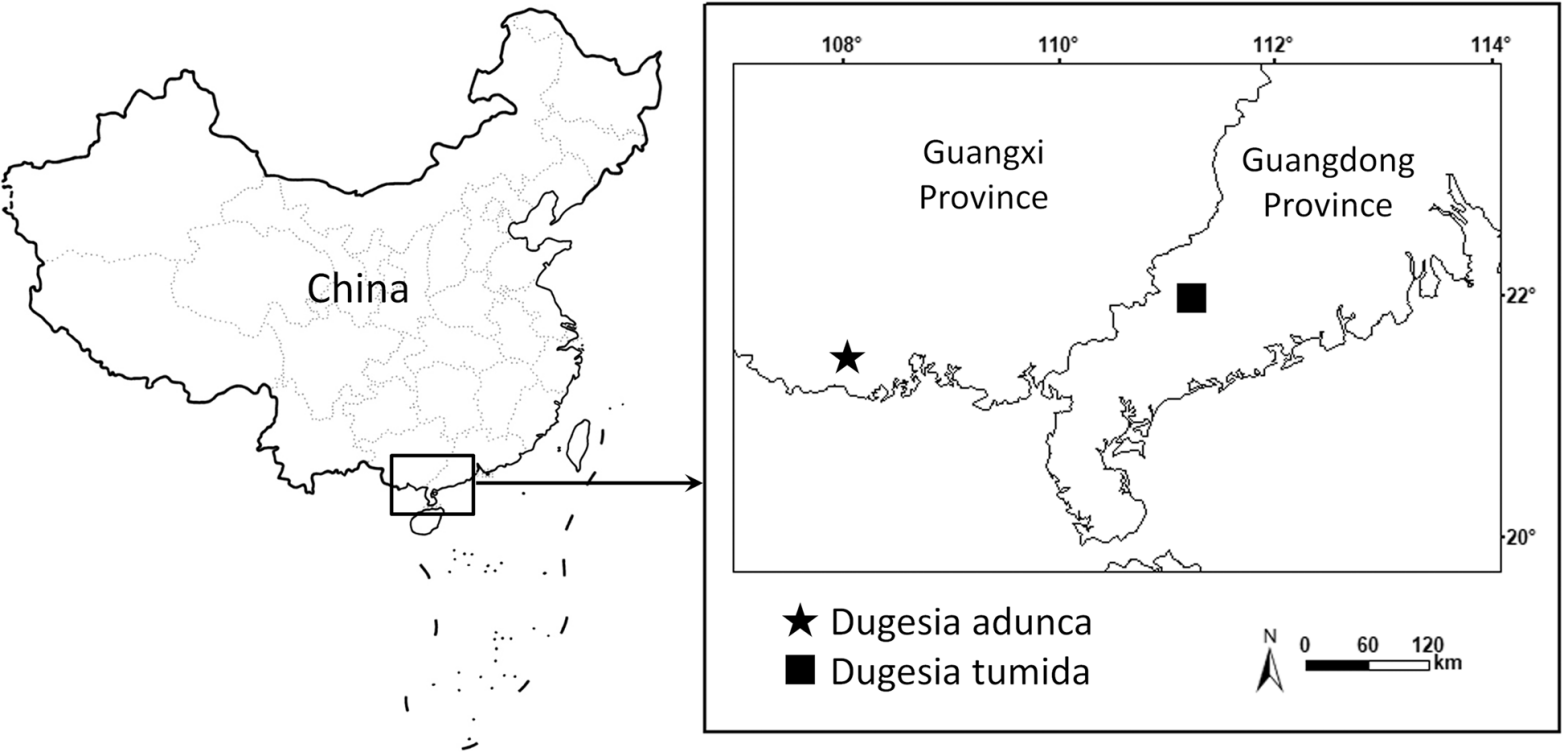
Collection sites of *Dugesia* in Guangxi and Guangdong Province

**Fig. 3:**
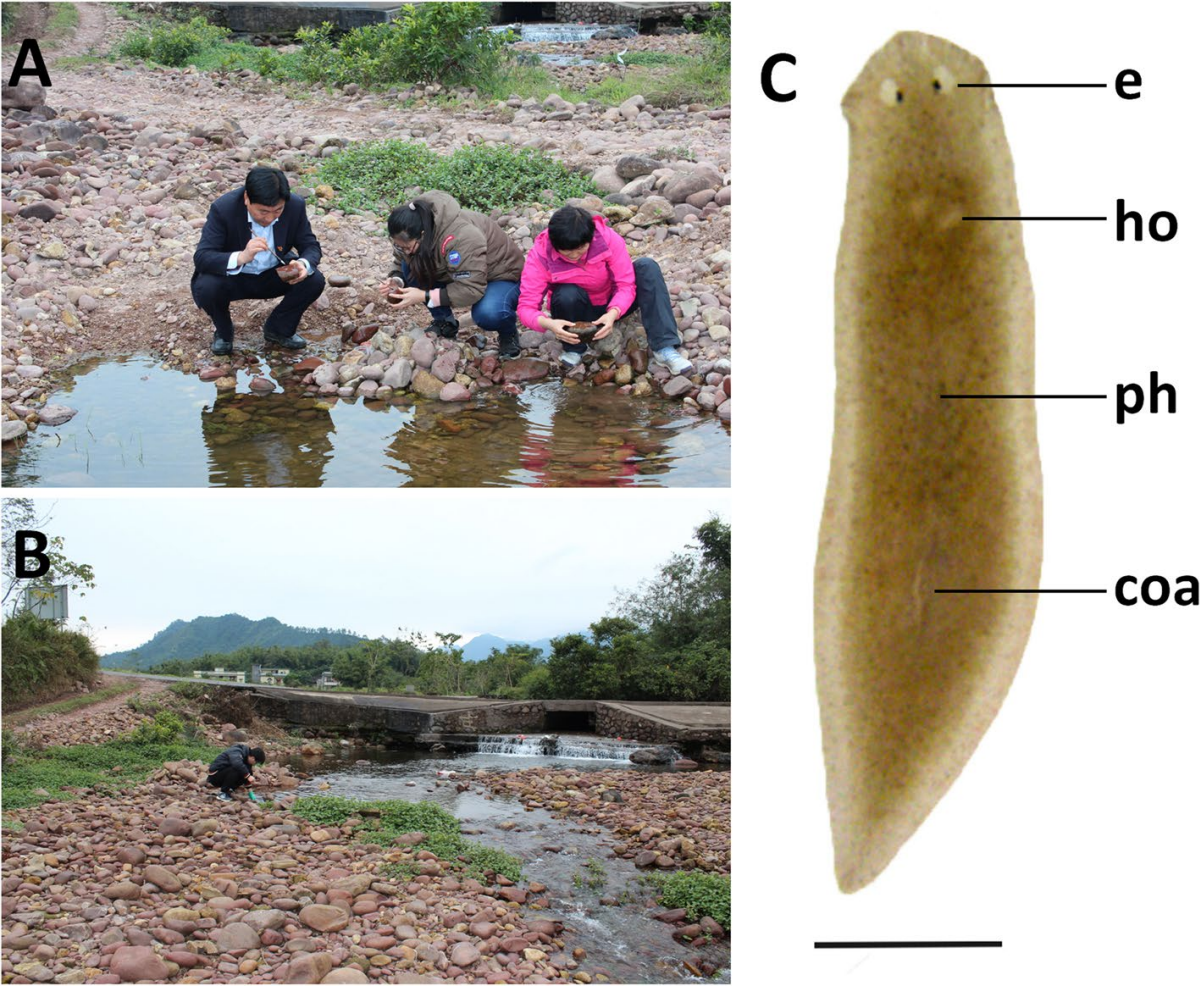
Habitat and external appearance of *Dugesia adunca*. **A**, **B** sampling site and habitat. **C** sexually mature, live individual, Scale bar: 2 mm. Abbreviations: coa, copulatory apparatus; e, eye; ho, hyperplasic ovary; ph, pharynx

On 31 December 2018, the specimens were collected from a pond in a riverbed on the Shiwan Dashan Mountain in Guangxi Province (Figs. 2, 3A, B), at an altitude of 81 m above sea level (a.s.l.), and at an air temperature of 14 °C; water temperature was higher, viz., 23 °C.

### Material examined

Holotype: ZMHNU-SMG9, Shimengou village (21°49′22"N 107°59′34''E; alt. 81 m a.s.l.), Fangcheng County, Guangxi Province, China, 31 December 2018, coll. G-W Chen, Z-M Dong and co-workers, sagittal sections on 20 slides.

Paratypes: ZMHNU-SMG1-4, 7, 8, ibid., sagittal sections on 12, 11, 22, 12, 8 and 20 slides, respectively; ZMHNU-SMG10, ibid., horizontal sections on 25 slides; ZMHNU-SMG 11 and 12, ibid., transverse sections on 19 and 18 slides, respectively; RMNH VER. 19981.a, ibid., sagittal sections on 7 slides; RMNH VER. 19981.b, ibid., sagittal sections on 11 slides.

### Diagnosis

*Dugesia adunca* is characterized by the presence of the following features: live, mature animals rather small; asymmetrical openings of the oviducts into the bursal canal; penis papilla with shape of an aquiline bill, albeit with a blunt tip; asymmetrical penis papilla, with a large antero-dorsal lip and a much smaller ventro-posterior lip; very large seminal vesicle, provided with trabeculae; small diaphragm; mixoploid karyotype with diploid complements of 2n = 2x = 16 and triploid complements of 2n = 3x = 24, with all chromosomes being metacentric.

### Etymology

The specific epithet is derived from the Latin adjective *aduncus*, hooked, aquiline, and alludes to the highly bent penis papilla.

### Karyology

**Fig. 4:**
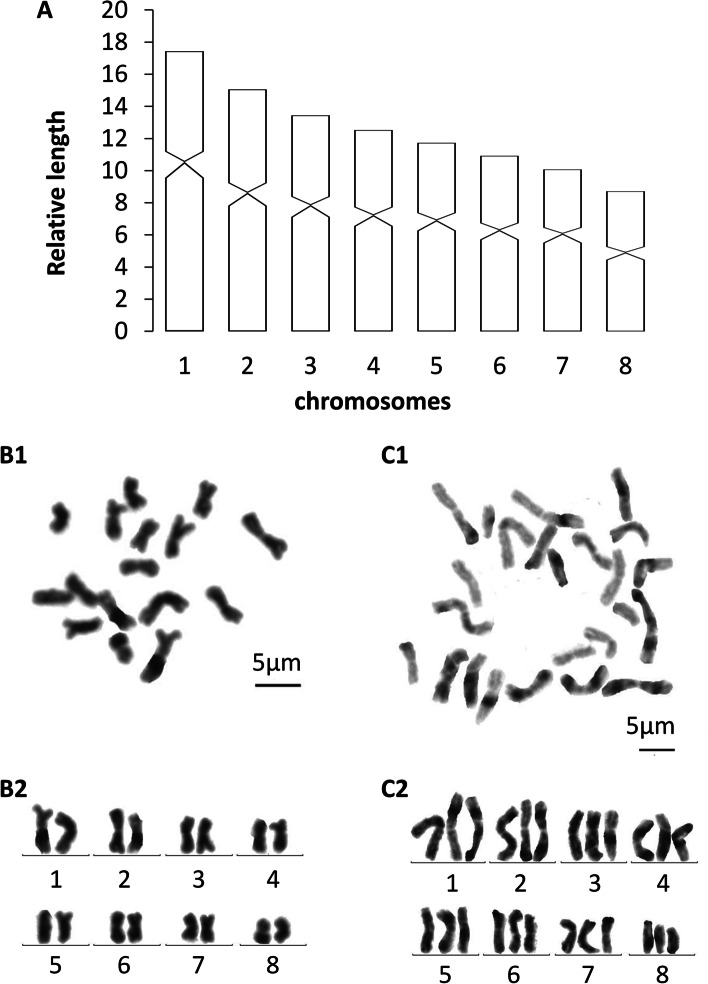
*Dugesia adunca*. **A** idiogram. **B1** metaphase plate of diploid complement. **B2** karyogram of diploid complement. **C1** metaphase plate of triploid set. **C2** karyogram of triploid set. Scale bar: 5 μm

Each of the seven randomly selected specimens that were used to examine the metaphase plates exhibited mixoploid chromosome complements. In a total of 100 metaphase plates, 45 exhibited a diploid chromosome portrait of 2n = 2x = 16, while 42 of the plates had a triploid complement of 2n = 3x = 24, with all chromosomes being metacentric (Fig. 4). In the remaining 13 metaphase plates, some lacked well-dispersed chromosomes, while others suffered from over-dispersed sets of chromosomes, which thus prevented proper determination of their karyotype. Karyotype parameters (relative length, arm ratio, centromeric index) are given in Table 2. The first pair of chromosomes is clearly larger than the others, being 1.99 times larger than the shortest one. A chromosomal plate and idiogram of the karyotype are shown in Fig. 4.

**Table 2:** Karyotype parameters (mean values and standard deviations) of *Dugesia adunca;* m: metacentric

Chromosome	Relative length	Arm ratio	Centromeric index	Chromosome type
1	17.43 ± 0.97	1.53 ± 0.34	41.34 ± 2.41	m
2	15.05 ± 0.58	1.35 ± 0.11	44.97 ± 1.78	m
3	13.44 ± 0.32	1.40 ± 0.18	41.86 ± 2.22	m
4	12.53 ± 0.50	1.37 ± 0.16	42.11 ± 1.53	m
5	11.73 ± 0.29	1.46 ± 0.17	44.77 ± 2.16	m
6	10.92 ± 0.56	1.37 ± 0.25	43.50 ± 3.28	m
7	10.07 ± 0.60	1.53 ± 0.34	42.43 ± 2.46	m
8	8.72 ± 0.82	1.28 ± 0.12	43.57 ± 0.94	m

### Description

Body of both asexual and sexual live specimens is quite small, the asexual animals measuring 5–7 mm in length and 1.3–1.6 mm in width, while the sexual worms are 8–10 mm in length and 1.7–2.0 mm in width. Low-triangular head provided with two blunt auricles and two eyes, which are placed in pigment-free spots (Fig. 3C). Each pigmented eyecup houses numerous photoreceptor cells. The dorsal surface is light brown (RAL 1024), excepting the pale, broad margin of the body; the ventral surface shows a brown hue (RAL 1015), which is paler than the dorsal colouration.

Pharynx situated in the mid-region of the body, measuring about 1/7^th^ of the body length (Fig. 3C). Mouth opening located at the posterior end of the pharyngeal pocket. Outer pharyngeal musculature is composed of a subepithelial and thin layer of longitudinal muscles, followed by a thin layer of circular muscles; no extra inner layer of longitudinal muscles was observed. The inner pharyngeal musculature consists of a subepithelial, thick layer of circular muscle, followed by a thin layer of longitudinal muscle.

**Fig. 5:**
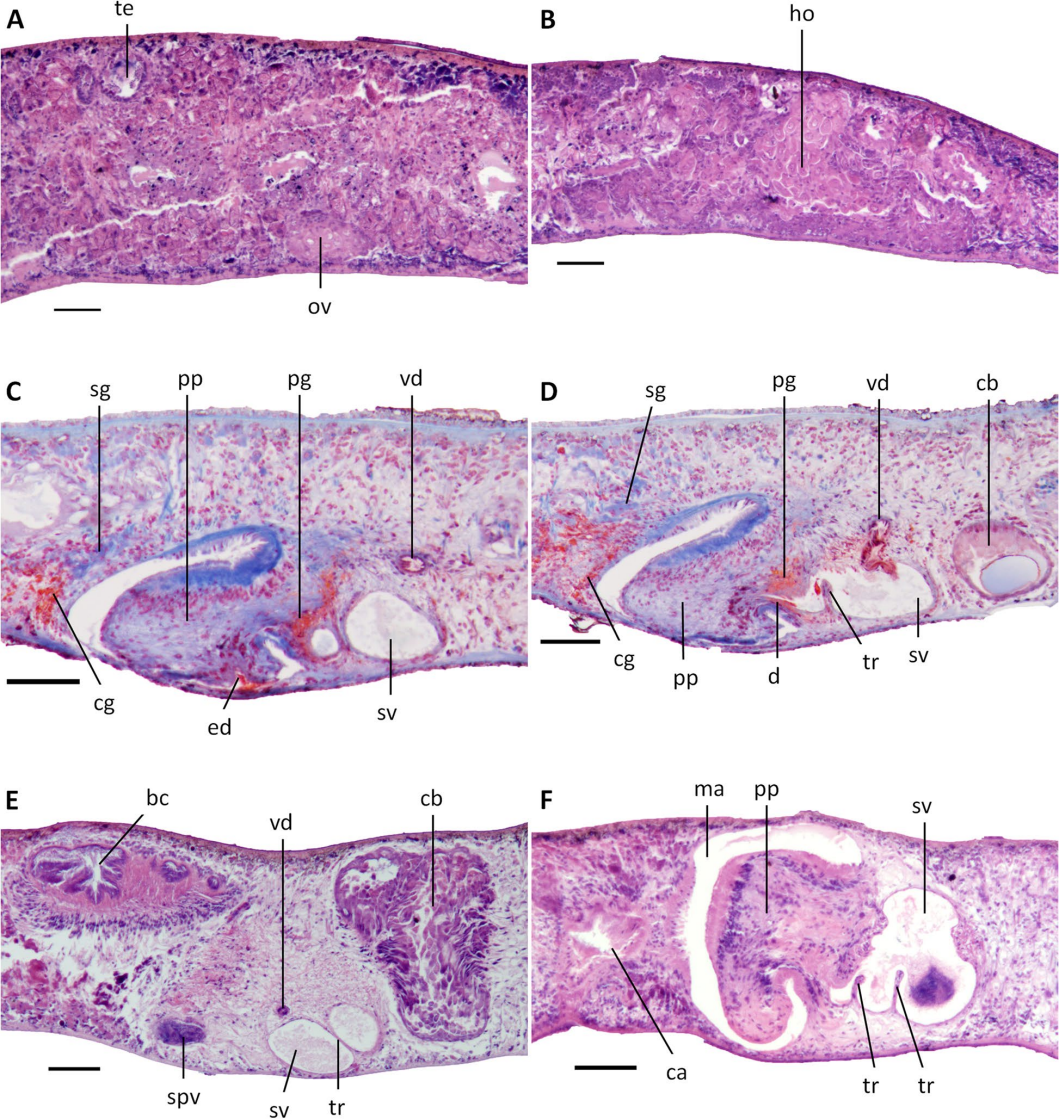
*Dugesia adunca*. **A** sagittal section of paratype SMG1, showing ovary and testes. **B** sagittal section of paratype SMG1, showing hyperplasic ovary. **C** sagittal section of paratype SMG8, showing cement glands, penis glands, and ventral opening of ejaculatory duct. **D** sagittal section of paratype SMG8, showing cement glands, penis glands and diaphragm. **E** sagittal section of holotype SMG9, showing large copulatory bursa and seminal vesicle with trabeculae. **F** sagittal section of paratype SMG4, showing the aquiline penis papilla and large seminal vesicle with trabeculae. Scale bars: 100 μm. Abbreviations: bc, bursal canal; ca, common atrium; cb, copulatory bursa; cg, cement glands; d, diaphragm; ed, ejaculatory duct; ho, hyperplasic ovary; ma, male atrium; ov, ovary; pg, penis glands; pp, penis papilla; sg, shell glands; spv, spermiducal vesicle; sv, seminal vesicle; te, testes; tr, trabecula; vd, vas deferens

In those specimens in which we were able to examine the ovaries, most of the gonads were hyperplasic (specimens SMG2, 6, 8, 10, 11 and 12), excepting specimens SMG1 and SMG3 (Fig. 5A, B). In general, the hyperplasic ovaries occupy more than one-half of the dorso-ventral space or even the entire dorso-ventral space, with several scattered masses situated at a short distance behind the brain. In live animals, such ovaries are already visible from the dorsal side of the body as two small whitish patches (Fig. 3C). From the ovaries, the oviducts run ventrally in a caudal direction to the level of the genital pore, after which they curve dorso-medially to open separately and asymmetrically into the descending portion of the bursal canal, near its communication with the common atrium; the right oviducal branch opens dorsally to the left one (Fig. 7). The oviducts are lined with an infranucleated epithelium.

The well-developed testes are situated dorsally and provided with mature spermatozoa. Testicular follicles extend from the posterior level of the ovaries to almost the posterior end of the body.

**Fig. 6:**
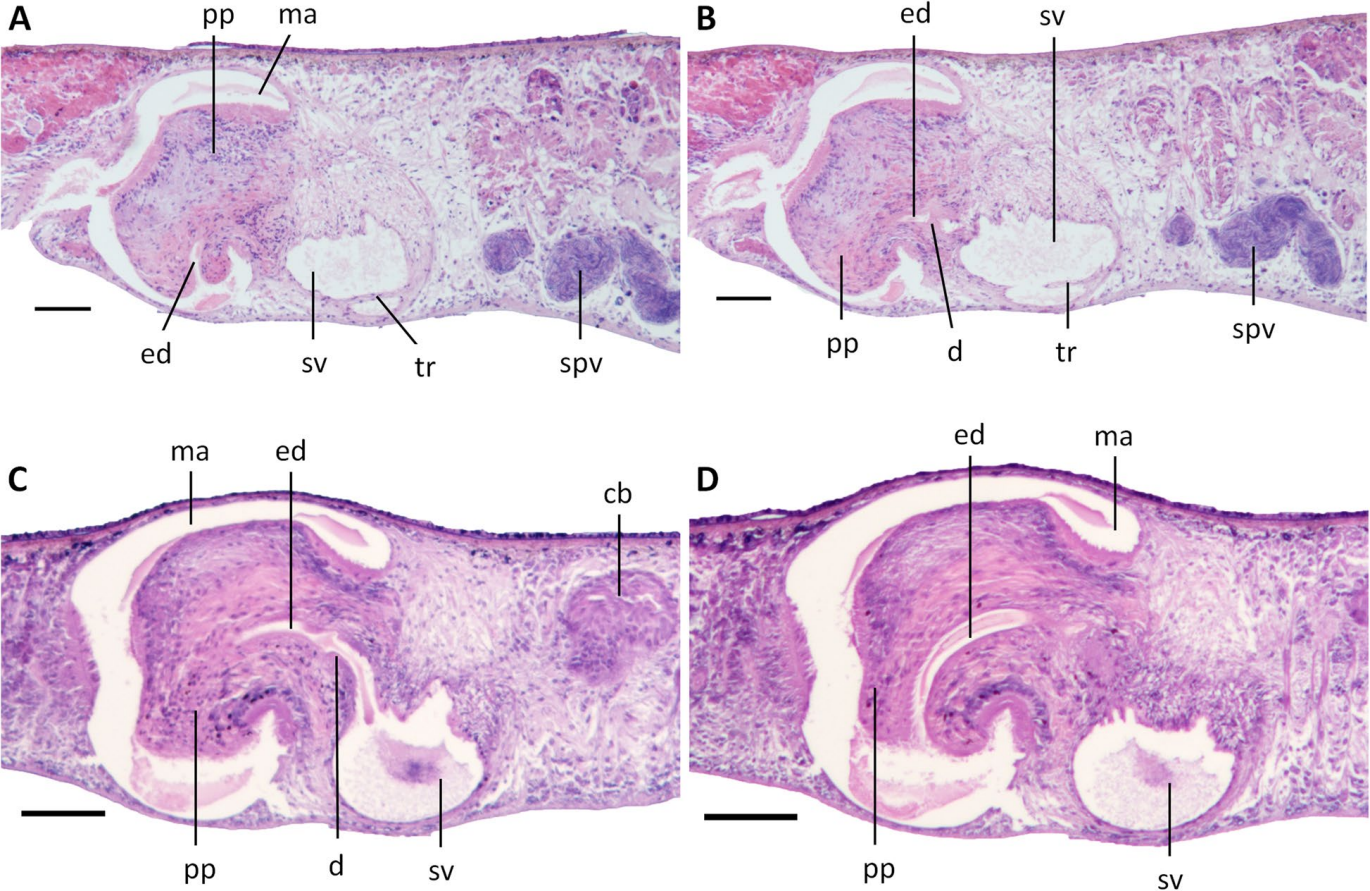
*Dugesia adunca*. **A** sagittal section of holotype SMG9, showing ventral opening of ejaculatory duct, aquiline penis papilla, and seminal vesicle with trabeculae. **B** sagittal section of holotype SMG9, showing ejaculatory duct, diaphragm, aquiline penis papilla, and large seminal vesicle. **C** sagittal section of paratype SMG3, showing ejaculatory duct, diaphragm, and aquiline penis papilla. **D** sagittal section of paratype SMG3, showing ejaculatory duct, and aquiline penis papilla. Scale bars: 100 μm. Abbreviations: cb, copulatory bursa; d, diaphragm; ed, ejaculatory duct; ma, male atrium; pp, penis papilla; spv, spermiducal vesicle; sv, seminal vesicle; tr, trabecula

**Fig. 7:**
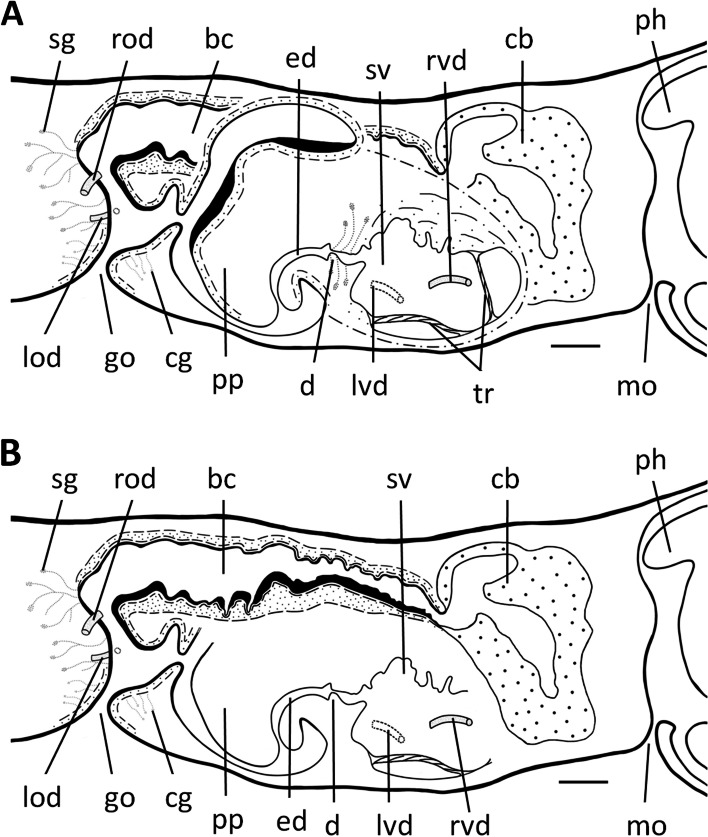
*Dugesia adunca*. Sagittal reconstruction of the copulatory apparatus of holotype SMG9. **A** sagittal reconstruction of male copulatory apparatus. **B** sagittal reconstruction of female copulatory apparatus. Scale bar: 100 μm. Abbreviations: bc, bursal canal; cb, copulatory bursa; cg, cement glands; d, diaphragm; ed, ejaculatory duct; go, gonopore; lod, left oviduct; lvd, left vas deferens; mo, mouth; ph, pharynx; pp, penis papilla; rod, right oviduct; rvd, right vas deferens; sg, shell glands; sv, seminal vesicle; tr, trabecula

At the level of the copulatory bursa, the vasa deferentia expand to form spermiducal vesicles, which are packed with mature spermatozoa (Figs. 5E, 6A, B). Upon reaching the large penis bulb, the vasa deferentia quickly decrease considerably in diameter and, subsequently, penetrate the wall of the penis bulb to open separately into the seminal vesicle (Figs. 5C, D, 7). The approach and position of the actual openings of the vasa deferentia into the seminal vesicle differ somewhat between the specimens examined. In the holotype SMG-9 the ducts open more or less into the mid-lateral portions of the vesicle, while, for example, in specimen SMG-8 the sperm ducts approach the vesicle from the dorsal side and thus open into the mid-dorsal section of the seminal vesicle. The sperm ducts are lined with a nucleated epithelium and surrounded by a layer of circular muscles.

The voluminous, sac-shaped seminal vesicle occupies the major portion of the penis bulb and thus may occupy one-half or even more of the dorso-ventral space (Figs. 5F, 6B, C, 7A). As a consequence, the penis bulb is also a rather large structure, in size almost equalling the penis papilla.

The seminal vesicle is lined by a flat, nucleated epithelium and is provided with several trabeculae that, seemingly, partition off portions of the vesicle (Figs. 5E, F, 6A, B, 7A). The seminal vesicle is surrounded by intermingled muscle fibres, which on its dorsal side may form a loose coat of considerable thickness (Figs. 5D, 7A). A short duct connects the seminal vesicle with the diaphragm, the latter leading to the ejaculatory duct. The small diaphragm is located at the root and near the ventral side of the penis papilla and receives the abundant secretion of erythrophil penis glands (Figs. 5C, D, 6B, C, 7A). Shortly after its communication with the diaphragm, the relatively narrow ejaculatory duct abruptly exhibits a knee-shaped, ventrally directed bend, thus following the equally curved tip of the penis papilla. The ejaculatory duct opens at the blunt tip of the penis papilla, albeit that the opening is displaced in posterior direction, thus giving rise to a highly asymmetrical papilla with a large antero-dorsal lip and a much smaller ventro-posterior lip (Figs. 5C, D, 6, 7A). The penis papilla has a characteristic shape, in that from its root it immediately and sharply curves towards the ventral body surface by means of a knee-shaped bend, giving it the appearance of a hooked, aquiline bill; it is covered with an infranucleated epithelium, which is underlain by a subepithelial layer of circular muscle, followed by a layer of longitudinal muscle fibres.

The sac-shaped copulatory bursa occupies almost the entire dorso-ventral space and is lined by a columnar, vacuolated epithelium with basal nuclei and is devoid of any surrounding musculature (Figs. 5E, 7B). In specimens SMG-2 and SMG-8, the copulatory bursa contains remnants of spermatophore. Near its communication with the postero-dorsal section of the bursa, the bursal canal at first is rather narrow but then, while running posteriad, it quickly widens and thus may occupy about 1/4th of the dorso-ventral space (Figs. 5E, 7B). The bursal canal runs in a caudal direction to the left side of the male copulatory apparatus. The posterior section of the canal narrows again and exhibits an antero-ventrally directed, knee-shaped bend, after which it opens into the common atrium (Fig. 7). The bursal canal is lined with a cylindrical, infranucleated, ciliated epithelium that is thrown into large folds (Figs. 5E, 7B). The bursal canal is surrounded by a subepithelial layer of longitudinal muscles, followed by a layer of circular muscle that is particularly well developed on the ventral wall of the canal; an extra outer layer of longitudinal musculature, forming the ectal reinforcement, extends from the copulatory bursa to the atrium. Cyanophil shell glands open into the vaginal region of the bursal canal, near the oviducal openings (Figs. 5C, D, 7).

The genital atrium is divided into a common atrium and male atrium, which communicate via a constriction. The common atrium communicates with a gonoduct, which leads to the ventral gonopore; the gonoduct is lined by a columnar epithelium and receives the openings of abundant cement glands (Figs. 5C, D, 7).

## Reproduction

Only five animals were sexually mature at collection. After about 1 month of rearing under laboratory conditions, 15 asexual specimens sexualized and presented hyperplasic ovaries. These sexualized animals did not produce any cocoons, in contrast to the naturally sexual specimens, which produced a total of 10 cocoons. The spherical cocoons (0.8 mm in diameter) were dark brownish and provided with a stalk. After about 4–6 days after deposition, juvenile worms hatched from the cocoons. These juvenile planarians were light brown, after about 3–5 days measuring 1.5–2 mm in length and 0.3 mm in width. The juveniles grew slowly, reaching maturity after about 6 months, then measuring 6–8 mm in length and 1.2 mm in width. During the laboratory culture, more than 12 juveniles became mature, sexualized animals, in possession of hyperplasic ovaries; none of these worms produced any cocoons.

## Comparative discussion

With respect to the opening of the ejaculatory duct at the penis papilla, two character states have been recognized in species of *Dugesia*, viz., terminal and subterminal openings (cf. [27]). Subterminal openings are generally located on the ventral side of the penis papilla (cf. [27], Fig. 6C), but three species form an exception to this rule, as in *D. hepta* Pala et al., 1981 the opening is lateral [28], while in *D. umbonata* Song & Wang, 2020 and *D. majuscula* the subterminal opening is located at the dorsal side [2, 4], as may be the case also in some specimens of *D. tumida* (see below).

At first sight, one may be inclined to classify the opening of the ejaculatory duct in *D. adunca* as simply being subterminal. However, this would fail to take into account also that, generally, subterminal openings occur in penis papillae that are distinctly cylindrical or barrel-shaped. In contrast, in *D. adunca* the penis papilla has the shape of hooked bill, albeit with a blunt tip. There are only a few species that share with *D. adunca* such a highly aquiline shape of the penis papilla, viz., *D. absoloni* (Komàrek, 1919), *D. aethiopica* Stocchino, Corso, Manconi & Pala, 2002, *D. astrocheta* Marcus, 1958, *D. nannophallus* Ball, 1970, and perhaps also *D. superioris* Stocchino & Sluys, 2013. However, *D. adunca* cannot be synonymized with any of these species. For example, in *D. absoloni* the copulatory bursa connects with the gut, while genito-intestinal connections are absent in *D. adunca*. The latter differs from *D. aethiopica* in its molecular composition (see Fig. 1), karyology (*D. aethiopica*: haploid number n = 9), and, for example, the openings of the oviducts into the bursal canal (symmetrical in *D. aethiopica*) and the presence of a parenchymatic cavity on the penis papilla of *D. aethiopica*. *D. astrocheta* and *D. nannophallus* lack the large penis bulb as well as the voluminous seminal vesicle that are present in *D. adunca*, while in *D. nannophallus* the oviducts open symmetrically into the bursal canal. In *D. superioris* the ejaculatory duct follows a dorsally displaced course through the penis papilla, in contrast to the ventrally displaced trajectory in *D. adunca*, while the former exhibits a triploid chromosome complement of 24 + 1 B-chromosomes, with a haploid number of n = 8, in contrast to the chromosome portrait of *D. adunca*, which shows a mixoploid karyotype with diploid complements of 2n = 2x = 16 and triploid sets of 2n = 3x = 24.

A large seminal vesicle is not uncommon among species of *Dugesia*, but for no species the presence of distinct trabeculae has been reported, as present in the vesicle of *D. adunca*.

### *Dugesia tumida* Chen & Sluys, sp. nov.

Zoobank registration: urn:lsid:zoobank.org:act:B996992A-D06A-4C58-9400-F9720E1EC027.

### Collection site and habitat

**Fig. 8:**
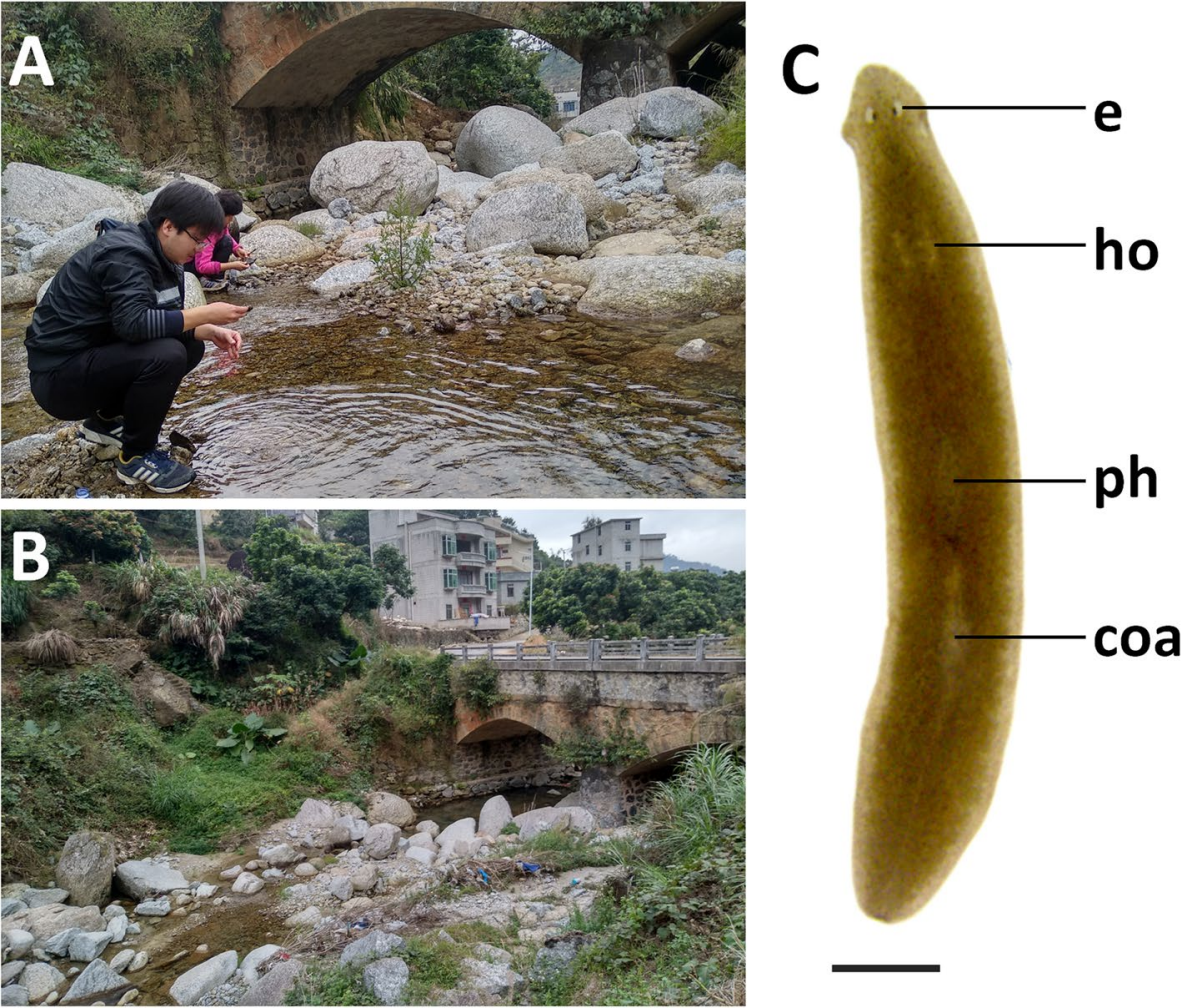
Habitat and external appearance of *Dugesia tumida*. **A**, **B** sampling site and habitat. **C** sexually mature, live individual, Scale bar: 2 mm. Abbreviations: coa, copulatory apparatus; e, eye; ho, hyperplasic ovary; ph, pharynx

On 4 January 2019, the specimens were collected from a stream on the Yunwu Mountain, Guangdong Province (Figs. 2, 8A, B), at an altitude of 558 m a.s.l.; air temperature was 19.5℃, and water temperature 16.5℃.

### Material examined

Holotype: ZMHNU-YLKB5, Youlukeng village (22°16′55"N 111°10′26''E), alt 558 m a.s.l., Xinyi County, Guangdong Province, China, 4 January 2019, coll. G-W Chen, Z-M Dong and co-workers, sagittal sections on 34 slides.

Paratypes: ZMHNU-YLKB1, 2, 4, 6, ibid., sagittal sections on 23, 17, 30, 34, and 20 slides, respectively; ZMHNU-YLKB8, ibid., transverse sections on 36 slides; ZMHNU-YLKB9, ibid., horizontal sections on 14 slides; RMNH VER. 19982.a, ibid., sagittal sections on 26 slides; RMNH VER. 19982.b, ibid., sagittal sections on 19 slides.

### Diagnosis

*Dugesia tumida* is characterized by the following features: penis papilla provided with a large, symmetrical penial valve from the middle of which arises the small, distal section of the papilla; a duct intercalated between the seminal vesicle and the small diaphragm; ventrally displaced ejaculatory duct curving upwards before opening to the exterior; penis papilla highly asymmetrical, having a slim and long ventral portion and a short and stubby dorsal part; vasa deferentia separately opening into antero-dorsal portion of seminal vesicle; oviducts openings symmetrically into ventral portion of the bursal canal, near its opening into the atrium; mixoploid karyotype, with diploid chromosome portraits of 2n = 2x = 16, and triploid complements of 2n = 3x = 24, with all chromosomes being metacentric.

### Etymology

The specific epithet is derived from the Latin adjective *tumidus*, swollen, and alludes to the fact that the root of the penis papilla is inflated to give rise to a penial valve.

### Karyology

**Fig. 9:**
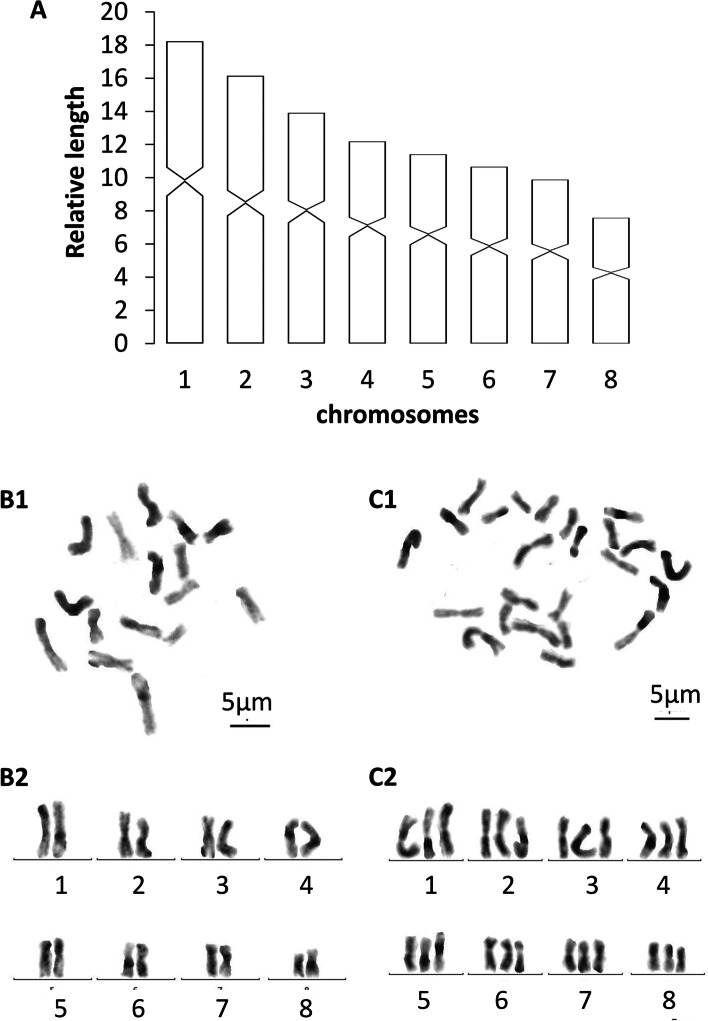
*Dugesia tumida*. **A** idiogram. **B1** metaphase plate of diploid complement. **B2** karyogram of diploid set. **C1** metaphase plate of triploid set. **C2** karyogram of triploid complement. Scale bar: 5 μm

Each of the five randomly selected specimens showed mixoploid chromosome complements. In a total of 100 metaphase plates, 34 exhibited diploid chromosome portraits of 2n = 2x = 16, while 51 of the plates had a triploid complement of 2n = 3x = 24, with all chromosomes being metacentric (Fig. 9). In the rest of 15 metaphase plates, some lacked well-dispersed chromosomes, while others had over-dispersed sets chromosomes, thus preventing adequate assessment of their karyotype. Karyotype parameters (relative length, arm ratio, centromere index) are given in Table 3. The first pair of chromosomes is clearly larger than the others, being 2.40 times larger than the shortest one. A chromosomal plate and idiogram of the karyotype are shown in Fig. 9.

**Table 3:** Karyotype parameters (mean values and standard deviations) of *Dugesia tumida;* m: metacentric

Chromosome	Relative length	Arm ratio	Centromeric index	Chromosome type
1	18.22 ± 0.98	1.17 ± 0.11	46.29 ± 2.21	m
2	16.15 ± 1.12	1.12 ± 0.12	47.48 ± 2.57	m
3	13.91 ± 0.49	1.37 ± 0.29	42.89 ± 4.44	m
4	12.18 ± 0.63	1.41 ± 0.31	42.32 ± 5.01	m
5	11.41 ± 0.39	1.37 ± 0.17	42.80 ± 2.96	m
6	10.66 ± 0.43	1.23 ± 0.12	45.23 ± 2.72	m
7	9.88 ± 0.55	1.3 ± 0.17	43.82 ± 3.06	m
8	7.58 ± 1.40	1.29 ± 0.07	43.86 ± 1.29	m

### Description

Live, asexual animals measured 8–11 mm in length and 1.0–1.5 mm in width, while sexualized worms were 18–32 mm in length and 1.9–2.2 mm in width. Triangular head provided with two blunt auricles and two eyes that are set in pigment-free spots. Each pigmented eyecup houses numerous photoreceptor cells. The dorsal body surface is brownish (RAL 1027), excepting the pale margin and the auricular grooves; the ventral surface (RAL 1014) is paler than the dorsal one (Fig. 8C).

Pharynx situated in the mid-region of the body, measuring about 1/6^th^ of the body length (Fig. 8C). Mouth opening located at the posterior end of the pharyngeal pocket. Outer pharyngeal musculature is composed a subepithelial and thin layer of longitudinal muscles, followed by a thin layer of circular muscles; there is no extra inner layer of longitudinal muscles. The inner pharyngeal musculature consists of a subepithelial, thick layer of circular muscle, followed by a thin layer of longitudinal muscle.

**Fig. 10:**
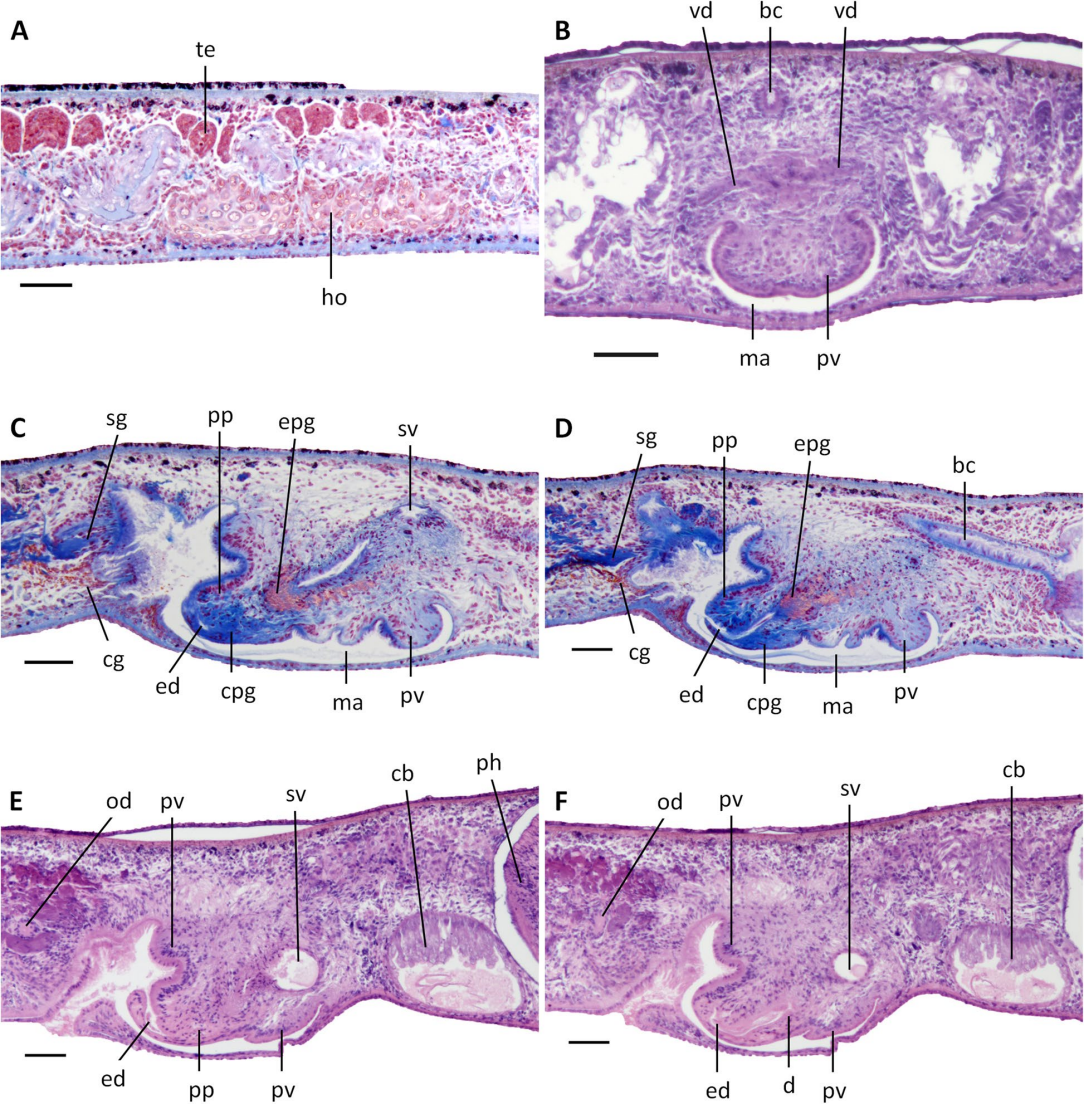
*Dugesia tumida*. **A** sagittal section of holotype YLKB5, showing hyperplasic ovary and testes. **B** transverse section of paratype YLKB8, showing the penial valve. **C** sagittal section of holotype YLKB5, showing cement glands, penis glands, penis papilla, and penial valve. **D** sagittal section of holotype YLKB5, showing cement glands, penis glands, penis papilla, dorsal opening of ejaculatory duct, and penial valve. **E** sagittal section of paratype YLKB2, showing penis papilla and dorsal opening of ejaculatory duct. **F** sagittal section of paratype YLKB2, showing penis papilla and diaphragm. Scale bars: 100 μm. Abbreviations: bc, bursal canal; cpg, cyanophil penis glands; d, diaphragm; ed, ejaculatory duct; epg, erythrophil penis glands; ho, hyperplasic ovary; ma, male atrium; od, oviduct; ph, pharynx; pp, penis papilla; pv, penial valve; sg, shell glands; sv, seminal vesicle; te, testes; vd, vas deferens

The hyperplasic ovaries occupy more than one-half of dorso-ventral space, with several scattered masses situated at a short distance behind the brain. In live animals, the hyperplasic ovaries are visible from the dorsal side of the body as two short whitish stripes (Fig. 8C). From the ovaries, the oviducts run ventrally in a caudal direction to the level of the genital pore, after which they curve dorso-medially to open separately and symmetrically into the ventral portion of the bursal canal, close to its communication with the atrium (Figs. 10E, F, 12, 13).

**Fig. 11:**
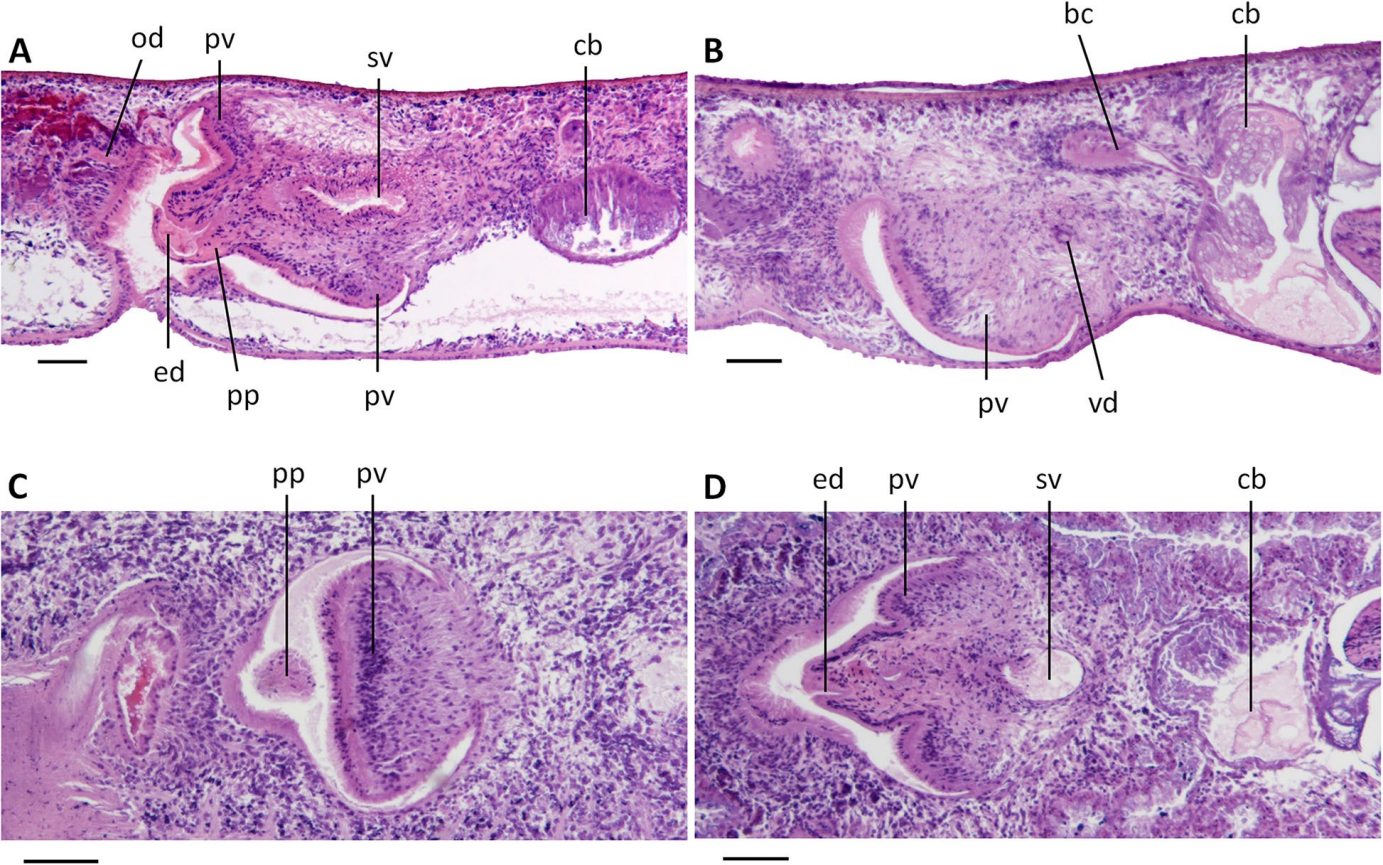
*Dugesia tumida*. **A** sagittal section of paratype YLKB1, showing penial valve. **B** sagittal section of paratype YLKB2, showing penial valve and copulatory bursa. **C** horizontal section of paratype YLKB9, showing penial valve. **D** horizontal section of paratype YLKB9, showing penial valve and small conical, distal portion of penis papilla. Scale bars: 100 μm. Abbreviations: bc, bursal canal; cb, copulatory bursa; ed, ejaculatory duct; od, oviduct; pp, penis papilla; pv, penial valve; sv, seminal vesicle; vd, vas deferens

**Fig. 12:**
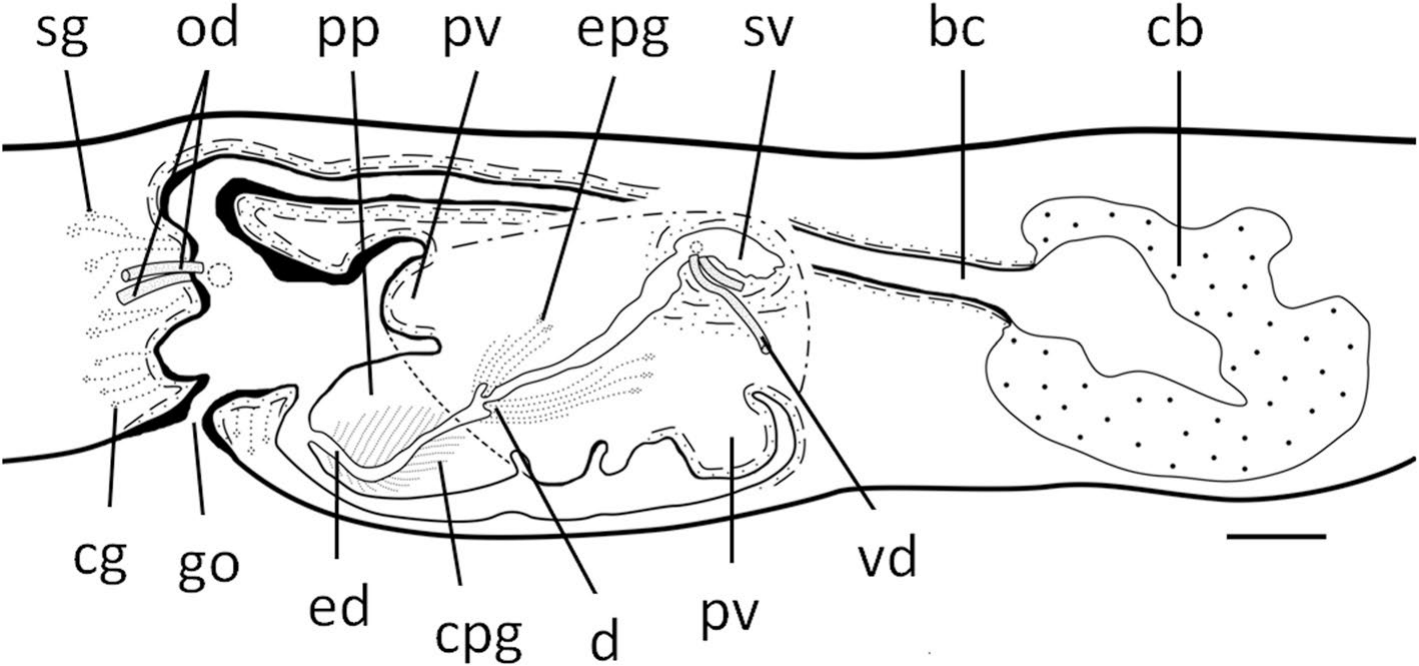
*Dugesia tumida*. Sagittal reconstruction of the copulatory apparatus of holotype YLKB5. Scale bar: 100 μm. Abbreviations: bc, bursal canal; cb, copulatory bursa; cg, cement glands; cpg, cyanophil penis glands; d, diaphragm; ed, ejaculatory duct; epg, erythrophil penis glands; go, gonopore; od, oviduct; pp, penis papilla; pv, penial valve; sg, shell glands; sv, seminal vesicle; vd, vas deferens

**Fig. 13:**
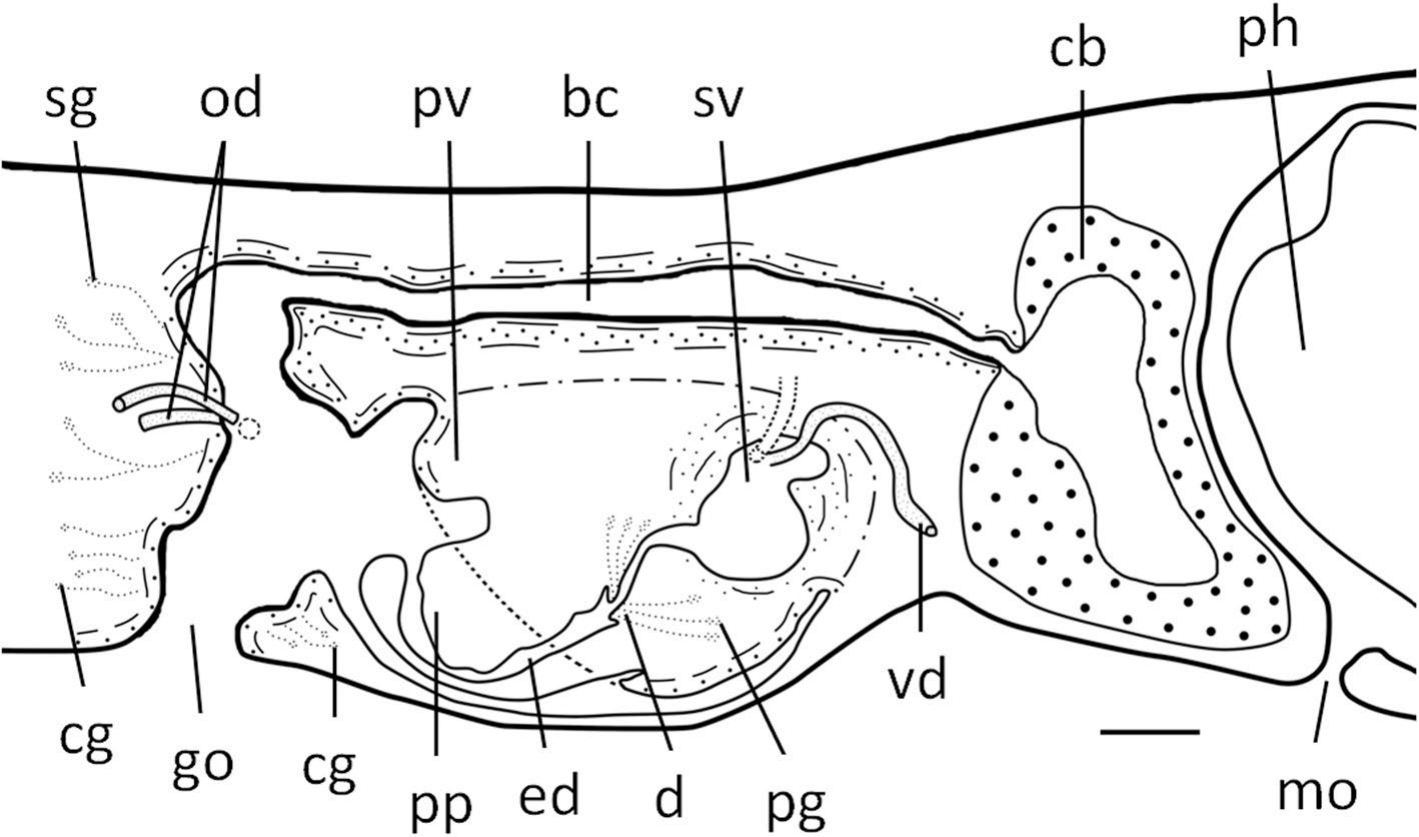
*Dugesia tumida*. Sagittal reconstruction of the copulatory apparatus of paratype YLKB2. Scale bar: 100 μm. Abbreviations: bc, bursal canal; cb, copulatory bursa; cg, cement glands; d, diaphragm; ed, ejaculatory duct; go, gonopore; mo, mouth; od, oviduct; pg, penis glands; ph, pharynx; pp, penis papilla; pv, penial valve; sg, shell glands; sv, seminal vesicle; vd, vas deferens

The poorly developed testes are situated dorsally, extending from the posterior level of the ovaries to almost the posterior end of the body; the follicles are devoid of mature spermatozoa (Fig. 10A) and, thus, sperm is also absent from the vasa deferentia of all specimens examined. Upon reaching the level of the penis bulb, the vasa deferentia curve dorso-mediad and penetrate the lateral wall of the penis bulb to open separately and symmetrically into the antero-dorsal or postero-ventral section of the seminal vesicle (Figs. 10B, 12, 13). The sperm ducts are lined with nucleated cells and surrounded by a layer of circular muscle. The sac-shaped seminal vesicle is lined by a flat, nucleated epithelium and is surrounded by a layer of intermingled muscle fibres (Figs. 10C-F, 11A, D, 12, 13). The posterior section of the seminal vesicle gives rise to a well-developed and rather long duct that via a small diaphragm opens into the ejaculatory duct. The small diaphragm is located at about the middle of the penis papilla and receives the abundant secretion of erythrophil penis glands (Figs. 10C, D, 12, 13). The ejaculatory duct follows a ventrally displaced course through the penis papilla, after which it turns upwards to open at the tip of the papilla. The ejaculatory duct receives the abundant secretion of cyanophil penis glands (Figs. 10C, D, 12). Depending upon the specimen examined, this opening of the ejaculatory duct is positioned at the dorsal wall of the tip of the penis papilla, as in specimen YLKB2 (Figs. 10 E, F, 13), or it is located at the dorsal portion of the tip (Fig. 11A) or the ventral part of the tip (Figs. 10C, D, 12). However, the common denominator in these seemingly different kinds of openings and trajectories is that the ejaculatory duct always curves upwards and that there is a rather slim and long ventral portion of the penis papilla, in contrast to a short and stubby dorsal part, thus making the penis papilla highly asymmetrical.

The broad base of the penis papilla consists of a swollen portion forming a very large, symmetrical penial valve that occupies the major portion of the male atrium (Figs. 10B-F, 11, 12, and 13). From the middle of this penial valve springs forth the much smaller distal portion of the penis papilla (Fig. 11D). This tip of the penis papilla is covered with an infranucleated epithelium, which is underlain with a subepithelial layer of circular muscle, followed by a layer of longitudinal muscle fibres, but the penial valve is covered with a nucleated epithelium.

The large, sac-shaped copulatory bursa occupies almost the entire dorso-ventral space and is lined by a stratified, columnar, vacuolated epithelium provided with basal nuclei and is devoid of any surrounding musculature (Figs. 10E, F, 11B, D, 12, 13). After originating, more or less, from the mid-posterior wall of the bursa, the bursal canal runs in a caudal direction to the left side of the male copulatory apparatus. Hereafter, and dorsally to the common atrium, the bursal canal makes a ventrally directed knee-shaped bend, after which it communicates with the common atrium. The bursal canal has more or less the same diameter over its entire length and is lined with cylindrical, infranucleated, ciliated cells and is surrounded by a subepithelial layer of longitudinal muscles, followed by a layer of circular muscle. An extra outer layer of longitudinal musculature, forming the ectal reinforcement, extends from the atrium to halfway on the bursal canal. Cyanophil shell glands open into the vaginal region of the bursal canal around the separate oviducal openings, which are located at the most ventral section of the bursal canal, close to its connection with the common atrium (Figs. 10E, F, 12, 13); the oviducts are lined with a columnar, infranucleated epithelium.

The common atrium communicates with a gonoduct, which is lined by a columnar epithelium and receives the openings of erythrophil cement glands (Figs. 10E, F, 12, 13).

## Reproduction

At collection, all worms were asexual. However, after about 5 months of rearing under laboratory conditions, the asexual specimens continuously sexualized, thus giving rise to at least 12 mature individuals, which after 10 months produced two cocoons. The spherical cocoons (1.0 mm in diameter) were dark brownish and provided with a stalk. At the time of writing of the manuscript of this paper, no juveniles had hatched from these cocoons.

## Comparative discussion

One feature that sets *D. tumida* apart from most of its congeners is the presence of a large penial valve, which is symmetrical and of the parenchymatic type. A penial valve, either symmetrical or asymmetrical, has been reported for the following species: *D. batuensis*, *D. hymanae* (Sivickis, 1928), *D. indonesiana* Kawakatsu, 1973, *D. leporii* Pala, Stocchino, Corso & Casu, 2000, *D. ryukyuensis*, and *D. uenorum* Kawakatsu & Mitchell, 1995 [11, 27]. However, *D. tumida* cannot be synonymized with any of these species because it has an ejaculatory duct that curves upwards (in contrast to downward curving duct in *D. batuensis*; central, straight duct in *D. hymanae* and *D. uenorum*; subterminal, ventral opening in *D. indonesiana*; ventrally displaced opening in *D. leporii* and *D. ryukuyensis*), a long duct intercalated between seminal vesicle and diaphragm (duct absent in *D. indonesiana, D. leporii, D. uenorum*), and highly asymmetrical penis papilla (symmetrical in *D. hymanae* and *D. uenorum*).

Our morphological taxonomic evaluation is molecularly supported for at least the species *D. batuensis* and *D. ryukyuensis,* as these two species are not closely related to *D. tumida*. Furthermore, karyologically *D. tumida* differs also from several of the species mentioned above. In *D. batuensis* and *D. ryukyuensis* the haploid complement is n = 7 chromosomes, in contrast to the haploid set in *D. tumida*, which is n = 8. Although the basic chromosome number of both *D. leporii* and *D. indonesiana* consists of 8 chromosomes, the karyotype of the first-mentioned species shows 12 metacentric and 4 submetacentric chromosomes, while that of the latter comprises a triploid set of 24 metacentric chromosomes [29, 30], thus differing from the mixoploid karyotype of *D. tumida*.

## General discussion

Analysis of the COI marker resulted in rather well-resolved phylogenetic trees (Supporting Information Fig. S2). The result of ITS-1 analysis showed a similar general topology, albeit that the Malagasy clade formed the most basal branch in ITS-1 BI tree (Supporting Information Fig. S3).

In the phylogenetic analyses of the concatenated dataset, the ML and BI trees have similar topologies, excepting the Madagascan species (Supporting Information Fig. S1). Because the BI tree is based on a partition analysis of each gene and codon position, it should be the most reliable result. This implies also that for reliable ML trees more molecular markers should be included, such as 18S & 28S & ITS-1 & COI [2].

The topology of the molecular phylogenetic trees basically agrees with results from previous molecular phylogenetic analyses, especially in the position of the Afrotropical and Malagasy groups (with high support in BI trees, pp = 1.00), which usually form the basal clades in such trees (Fig. 1; e.g. [2, 11, 31]). The separate species status of *D. adunca* and *D. tumida* are supported by their separate branches receiving high support values in the phylogenetic trees (pp = 1.00 & bs = 86, pp = 1.00 & bs = 80, respectively; Fig. 1, Supporting Information Fig. S1-S3). Although *D. adunca* and *D. tumida* belong to different clades, they form part of a major clade comprising species from the Far East.

In view of its geographic location, one would expect *D. adunca* to be closely related to Chinese species of *Dugesia*, but, in contrast, it forms part of a clade of five species that comprises also species from India, Thailand, Malaya, Japan, and Australia. This clade received rather high support (pp = 1.00, bs = 92).

With respect to genetic distances among species of *Dugesia*, previous studies showed that the lowest COI interspecific distance usually ranges between 6-10%, while lowest ITS-1 distance varies between 1-7% (e.g. [8, 11, 21, 32]). In the present study, the lowest COI distance value between members of the genus *Dugesia* is 2.41 %, between *D. sigmoides* Stocchino & Sluys, 2017 and *D. gibberosa* Stocchino & Sluys, 2017, while the lowest ITS-1 distance value is 0.37 %, between *D. aethiopica* and *D. sicula* (Supporting Information Table S1, S2). The lowest COI distance values between the two new species *D. adunca* and *D. tumida* and their congeners are 14.15 % and 14.71 %, respectively, while the distance between the two new species is 20.80%. For the ITS-1 marker, the lowest distance values of the two new species and their congeners are 6.14% and 2.21 %, respectively, while the distance between the two new species is 9.77%. Up to now, in total eight *Dugesia* species have been reported in South China, including the two new species *D. adunca* and *D. tumida* and six other species (*D. circumcisa* Chen & Dong, 2021, *D. majuscula*, *D. semiglobosa* Chen & Dong, 2021, *D. sinensis*, *D. umbonata* and *D. verrucula*). The COI and ITS-1 distance values between South China species range between 14.71-22.07%, and 2.21-12.92%, respectively. Moreover, the COI distance value between *D. adunca* and *D. aethiopica* is 23.69%, and for ITS-1 is 18.38%. ITS-1 distance values between *D. tumida* & *D. ryukyuensis* and *D. tumida* & D. *batuensis* are 12.27% and 9.91%, respectively (COI sequences of *D. ryukyuensis* and D. *batuensis* were removed in order to obtain more accurate values for the genetic distances, due to the fact that their COI sequences are less than 600 bp). Thus, in view of the results obtained in earlier studies, both markers provide ample evidence that *D. adunca* and *D. tumida* are molecularly well-separated from each other, as well as from their congeners.

The new species *D. adunca* and *D. tumida* share their mixoploid chromosome complements with a basic number of eight chromosomes with *D. japonica*, *D. majuscula*, *D. semiglobosa* and *D. siamana* Kawakatsu, 1980 [4, 33, 34]. Most of these species show one particular kind of mixoploid karyotype, viz., a combination of diploid and triploid complements. An exception is formed by *D. japonica,* which exhibits three types of mixoploid karyotypes in different populations, viz., diploid & triploid, diploid & tetraploid, and diploid & triploid & tetraploid. It is noteworthy that both *D. adunca* and *D. japonica* are able to reproduce sexually, despite the fact that they have a mixoploid karyotype consisting of diploid and triploid complements.

It has been established for species of *Dugesia* that, generally, hyperplasic ovaries are the cause of infertility, as in such abnormal ovaries the oocytes are abnormal ([3, 4] and references therein). Frequently, this infertility is enhanced by poorly developed testes, or even the absence of testes. This is precisely the case in the new species *D. tumida*, as it exhibits hyperplasic ovaries as well as poorly developed testes and, thus, it is unsurprising that the specimens of this species produced inviable cocoons.

Interpretation of the reproductive modality of *D. adunca* is more complex, as there is a difference between specimens in the field and those reared in the laboratory. In specimens SMG1 and SMG3 we were unable to find hyperplasic ovaries just after the animals had been collected in the field (Fig. 5A). Furthermore, an unknown number of juveniles hatched from cocoons that were collected in the field (since the cocoons hatched during transport to the laboratory, we were unable to determine the precise number of hatchlings). In contrast, animals reared in our laboratory cultures presented hyperplasic ovaries and did not produce any cocoons, which is not unexpected for such sexualized specimens. The condition in the wild may conform to the situation that in some species of *Dugesia* (*D. arabica* Harrath & Sluys, 2013, *D. majuscula* and *D. semiglobosa*) mixoploid, ex-fissiparous animals are able to produce fertile cocoons, albeit that the number of juveniles hatching from such cocoons is lower (e.g. [4, 34–37]). Thus, it may be the case that in the field usually fissiparous animals occasionally become ex-fissiparous, i.e., grow a fully developed reproductive apparatus. Presumably, these polyploid, i.e., mixoploid, specimens of *D. adunca* represent examples of gonado-somato-mosaicism, with the gonads having a ploidy level and a chromosomal development enabling successful sexual reproduction (cf. [38]).

At this moment we can only speculate on the causal factors behind the onset of the sexualization process, both in the field and under laboratory conditions. It may be the case that changes in the external conditions, such as light or water temperature, induce sexualization in asexual animals. Previous studies showed that in species that usually live in a rather warm habitat, lower temperatures induced worms to become ex-fissiparous and to develop hyperplasic ovaries. Such populations usually failed to produce cocoons, or only laid sterile cocoons, as was the case in *D. majuscula* and *D. semiglobosa* [4]. A similar situation may obtain in *D. adunca*, as this species lives in ponds with rather warm water and high air temperature in winter (see above), while from May to October, the maximum average air temperature is always over 30 °C in Southern Guangxi. However, in our laboratory cultures water temperature was lower (20 °C) and, thus, may have promoted sexualization and hyperplasic ovaries of the animals.

## Conclusion

Molecular, morphological, and karyological markers show that the two populations examined from Southern China represent members of the genus *Dugesia* and constitute two new, distinct species.

## Supplementary Information

**Additional file 1.**

**Additional file 2.**

**Additional file 3.**

**Additional file 4**.

**Additional file 5.**

## Data Availability

Holotypes and paratypes of the two new species were deposited in the Zoological Museum of the College of Life Science of Henan Normal University, Xinxiang, China (ZMHNU), and Naturalis Biodiversity Center, Leiden, The Netherlands (RMNH). Newly generated DNA sequences were deposited in GenBank (Table 1).

## Abbreviations

a.s.l.: Above sea level
BI: Bayesian Inference
C: Celsius
COI: Cytochrome c oxidase subunit I
ITS-1: Internal transcribed spacer-1
ML: Maximum-likelihood
M: Metre
mm: Millimeter
μm: Micrometre
PCR: Polymerase chain reaction
RMNH: Naturalis Biodiversity Center, Leiden, The Netherlands
ZMHNU: Zoological Museum of the College of Life Science of Henan Normal University, Xinxiang, China
