# Supplementary figures and images for: Two new species of Dugesia (Platyhelminthes, Tricladida, Dugesiidae) from the subtropical monsoon region in Southern China, with a discussion on reproductive modalities
Source: BMC Zool. 2022 May 23;7:25. doi: 10.1186/s40850-022-00127-8 (PMC10126995; doi:10.1186/s40850-022-00127-8)

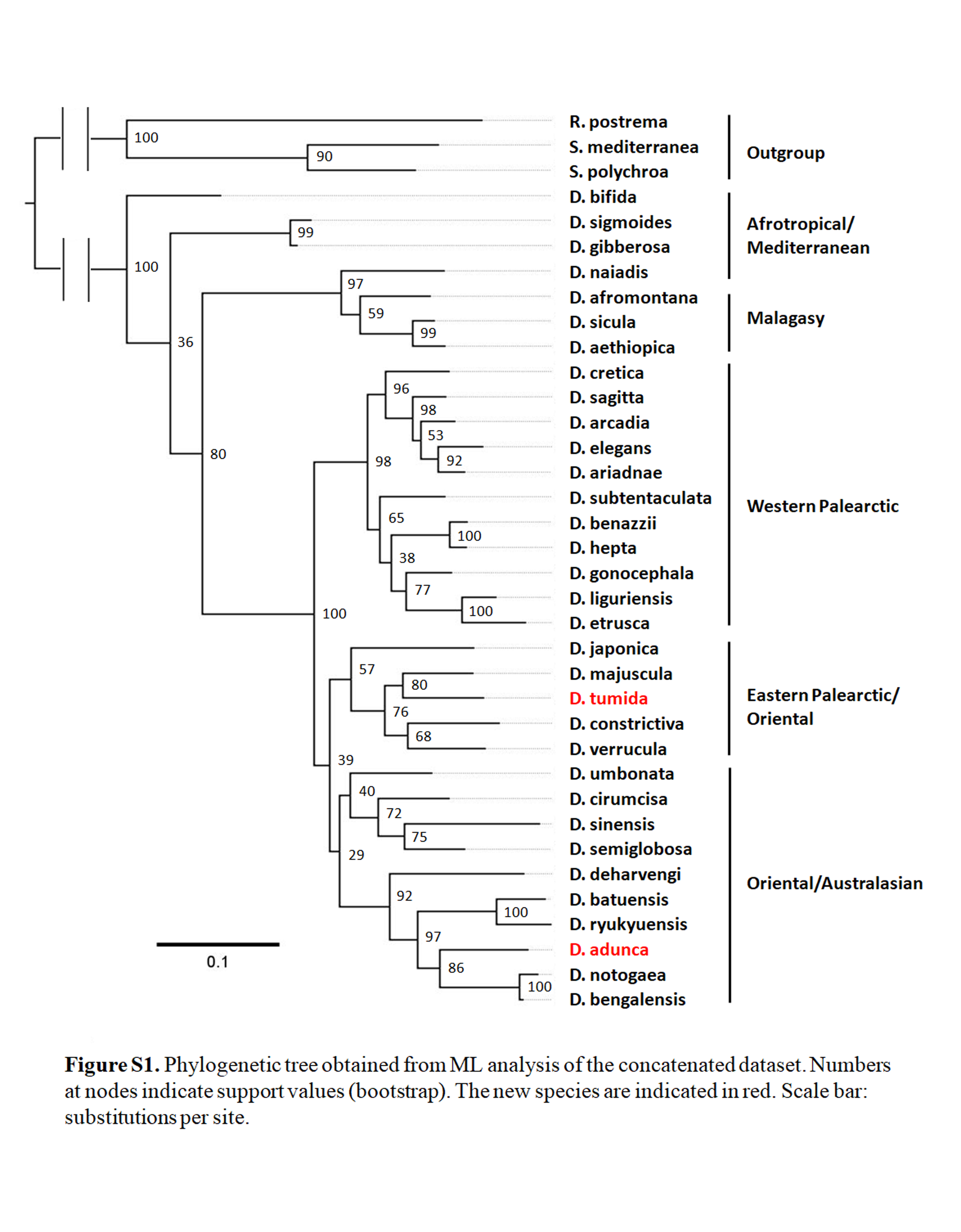

Supplement: Supplementary file 1 — Additional file 1. [file 40850_2022_127_MOESM1_ESM.tif]

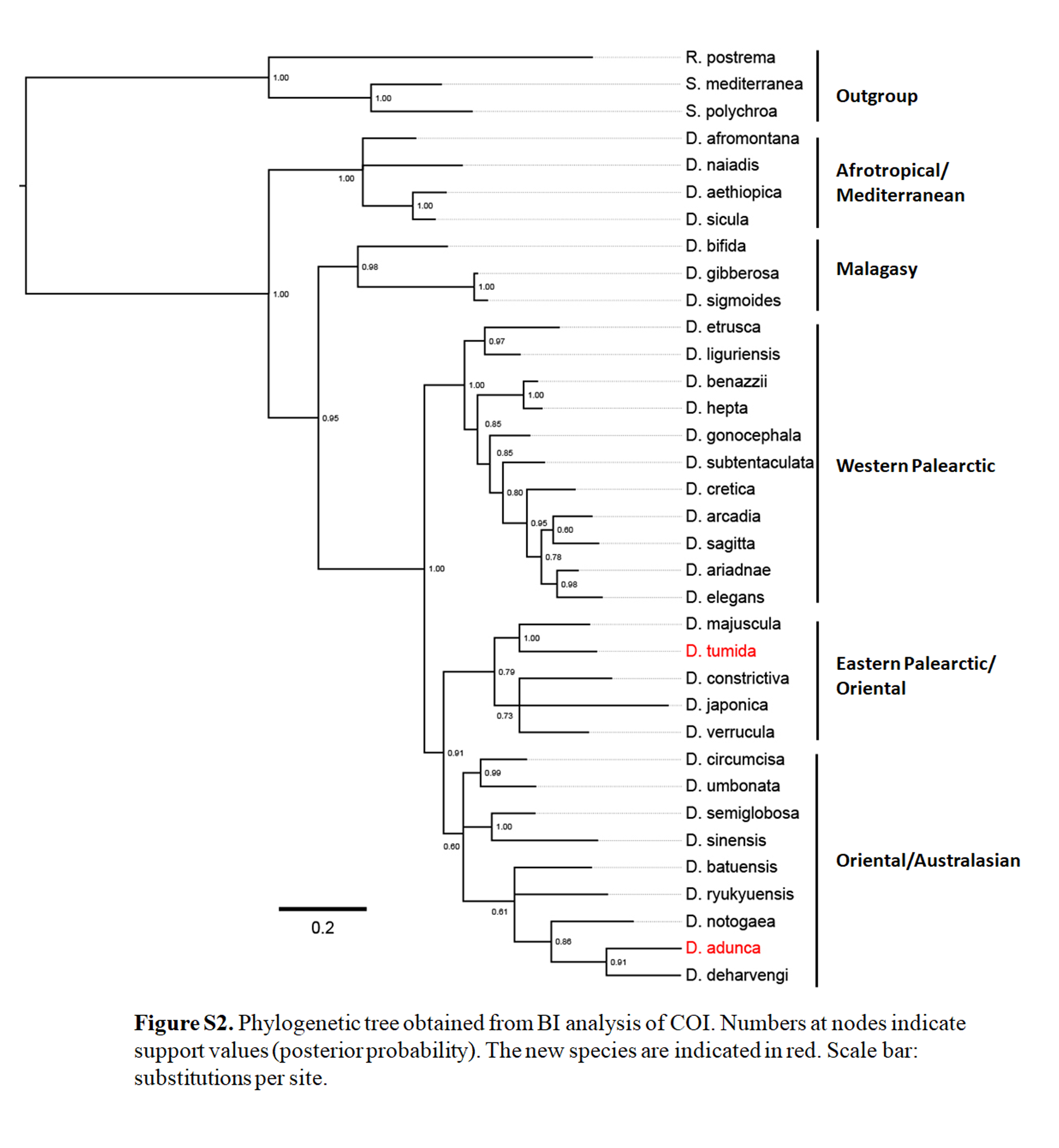

Supplement: Supplementary file 2 — Additional file 2. [file 40850_2022_127_MOESM2_ESM.tif]

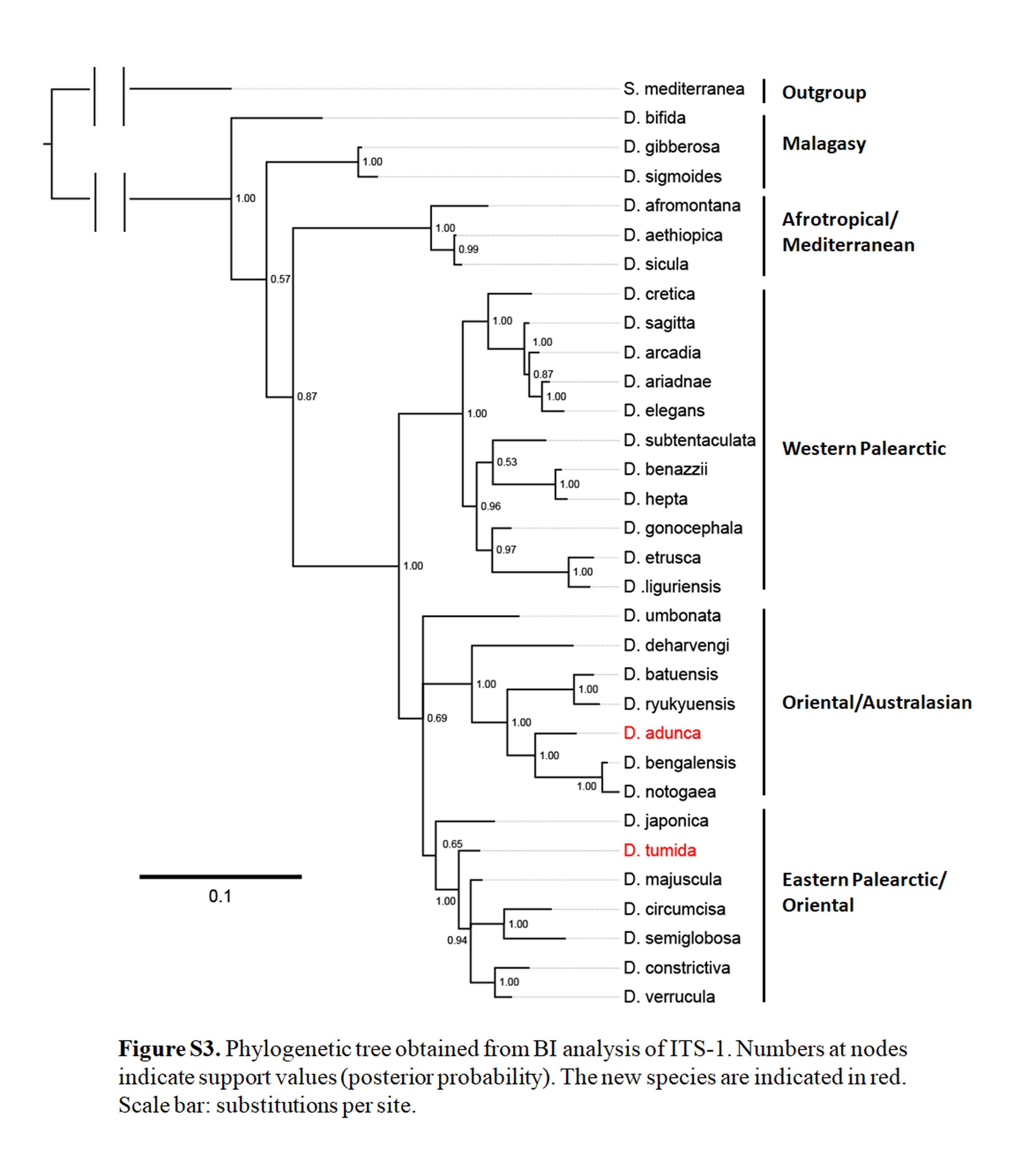

Supplement: Supplementary file 3 — Additional file 3. [file 40850_2022_127_MOESM3_ESM.tif]
